# Air-Mass Influence, Radiation, and Melt-Season Dynamics Jointly Shape Dissolved Organic Matter Composition and Radiocarbon Age in Glacier Outflows on the Tibetan Plateau

**DOI:** 10.1021/acs.est.6c06257

**Published:** 2026-08-05

**Authors:** Chen Huang, Mo Wang, Xiaolong Zhang, Frank-Thomas Koch, Christoph Gerbig, Hongxing Jiang, Baiqing Xu, Gerd Gleixner

**Affiliations:** † Department of Biogeochemical Processes, 28300 Max Planck Institute for Biogeochemistry, Jena 07745, Germany; ‡ State Key Laboratory of Tibetan Plateau Earth System, Environment and Resources, Institute of Tibetan Plateau Research, 74529 Chinese Academy of Sciences, Beijing 100101, China; § Department of Biogeochemical Signals, Max Planck Institute for Biogeochemistry, Jena 07745, Germany; ∥ Shanghai Key Laboratory of Air Quality and Environmental Health, Department of Environmental Science and Engineering, 12478 Fudan University, Shanghai 200433, China

**Keywords:** dissolved organic matter, ultrahigh-resolution mass spectrometry, radiocarbon, glacier meltwater, seasonality, photochemical processing, atmospheric transport, monsoon influence

## Abstract

Glacier melt increasingly delivers bioavailable yet radiocarbon-depleted dissolved organic matter (DOM) to headwaters, but controls on DOM molecular composition and radiocarbon signatures remain unclear for the Tibetan Plateau. We coupled ultrahigh-resolution mass spectrometry with Δ^14^C measurements for 30 samples from 16 glacier outflows, including three ablation-season time series across contrasting climatic settings. DOC concentrations were uniformly low, but DOM composition was highly heterogeneous. Multivariate analyses identified longitude and shortwave radiation as primary regional correlates, while marked differences among neighboring outlets indicated strong local controls. Seasonal DOM patterns varied by site: at the monsoon-influenced Rongbuk Glacier on the northern slope of Mount Everest, DOM shifted from lipid-rich to more lignin-, tannin-, and aromatic-enriched compositions as inorganic deposition indicators increased, consistent with enhanced allochthonous influence; however, Δ^14^C became more modern over the season. In contrast, the westerly-influenced, high-radiation Xiongcai Glacier showed comparatively stable DOM composition but elevated and dynamic peptide-like signals. Across the Δ^14^C subset (*n* = 23), Δ^14^C correlated negatively with lipid-like contributions, revealing an underappreciated “old-but-potentially labile” component. These results show that regional climate, local catchment setting, and melt-season progression jointly govern the export of mixtures of young and old, labile and compositionally complex DOM.

## Graphical abstract

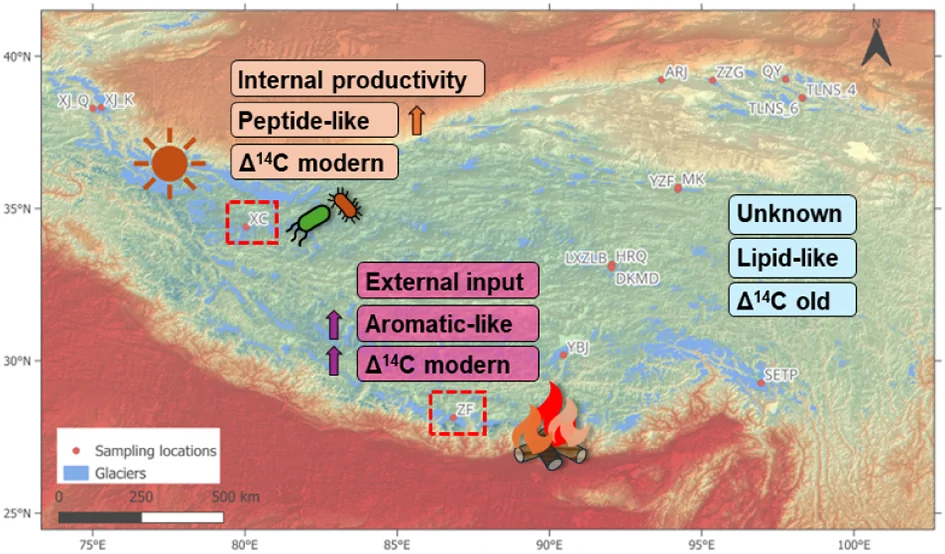

## 1 Introduction

Glacier runoff on the Tibetan Plateau is an essential headwater source for many Asian rivers and is highly sensitive to climate warming.^1,2^ In addition to supplying water resources, glacierized catchments export carbon and nutrients that can support downstream productivity and biogeochemical cycling.^3–5^ Within this exported carbon pool, dissolved organic matter (DOM) represents an important and reactive fraction, with previous estimates suggesting that dissolved organic carbon (DOC) export from Chinese mountain glaciers can reach 15.4 ± 6.1 Gg C yr^–1^.^5–7^ Glacier DOM is often enriched in aliphatic, lipid-like, and peptide-like compounds commonly associated with microbial production and lability,^8,9^ yet radiocarbon measurements from glacier systems, including those on the Tibetan Plateau, indicate that part of this DOM can be centuries to millennia old.^5,10^ This apparent contrast makes the Tibetan Plateau an important region for investigating how DOM molecular composition, potential lability, and carbon age are linked in glacier outflows.

Previous studies on the Tibetan Plateau show that glacier DOM is shaped by both in situ production and external inputs.^11–13^ Molecular analyses of snow, ice, snow pit, cryoconite, and glacier-fed waters have reported abundant lipid-, protein-, and CHON-rich formulas,^14–16^ consistent with microbial production and photochemical transformation processes that largely exist on the glacier surface.^17,18^ At the same time, atmospheric deposition can contribute terrestrial, combustion-derived, and anthropogenic organic matter.^10,11^ The combined influence of in situ production, atmospheric deposition, and strong photochemical processing is particularly relevant for the Tibetan Plateau, where glaciers are exposed to long-range transport from South Asia and westerly air masses, as well as to strong solar radiation at high elevation.^19–21^

These sources and processing pathways are expected to vary over the melt season as glacier hydrology evolves.^22,23^ Early-season runoff can be dominated by accumulated snowpack over the winter and baseflows with limited supraglacial connectivity, whereas later ablation can enhance the flushing of surface-derived material, microbial production, atmospheric deposits, and photochemically processed DOM.^23,24^ Such seasonal changes may alter both DOM molecular composition and radiocarbon signatures, but melt-season trajectories of glacier-outflow DOM remain poorly constrained on the Tibetan Plateau, particularly across catchments with contrasting monsoon and westerly influences.

Seasonal sources and processing pathways may also influence the relationship between DOM composition and radiocarbon age. Previous work on Tibetan Plateau glacier and glacier-fed stream DOM has shown old radiocarbon signatures, but the molecular characteristics associated with older versus younger DOM remain insufficiently resolved.^10^ This gap limits our ability to infer DOM source, transformation history, or carbon age from molecular composition alone. In particular, molecular signatures often associated with potential lability do not necessarily imply modern carbon, and old carbon may occur in chemically diverse pools, including lipid-like compounds from petrogenic or microbial-related sources and condensed aromatic-like fractions derived from combustion aerosols.^3,10,25–27^

The Tibetan Plateau provides a strong regional setting for testing how climatic gradients, source inputs, and melt-season processing jointly shape glacier DOM composition and carbon age.^1,6^ It spans arid, westerly-influenced regions and monsoon-influenced sectors with contrasting precipitation, radiation, and atmospheric transport pathways. Existing Tibetan Plateau studies have provided important insights into DOM in various glacier-related systems.^14,15,28^ However, few studies have integrated the effects of regional air-mass influence, radiation, melt-season evolution with ultrahigh-resolution molecular characterization and Δ^14^C constraints.

Here, we combine Orbitrap ultrahigh-resolution mass spectrometry (HR-MS) and Δ^14^C analysis to PPL solid-phase extracts to evaluate DOM composition and carbon age in glacier outflows across the Tibetan Plateau. We analyze a mid-ablation spatial data set of 16 glacier outflows together with three glacier-specific time series spanning the ablation season under contrasting climatic regimes. Specifically, we address three questions: (1) How do regional climatic gradients, including atmospheric circulation and solar radiation, structure glacier-outflow DOM composition across the Tibetan Plateau? (2) How does DOM composition evolve through the melt season in monsoon-influenced versus arid, westerly-influenced catchments? (3) How is molecular composition coupled to Δ^14^C, and which molecular features are associated with older or more modern carbon signatures?

## 2 Materials and Methods

### 2.1 Study Region and Sample Collections

Samples were collected from glacier outflows (proglacial streams) and one proglacial lake at or near glacier termini across the Tibetan Plateau between May and November 2022. The sites span a broad climatic and geographic gradient (39°48′–28°08′ N; 75°01′–96°56′ E) (Figure 1), with 5-year mean annual precipitation ranging from 128 ± 22 mm in the arid northwest (i.e., XJ_Q glacier) to 911 ± 236 mm in the humid southeast (i.e., SETP glacier). Site locations, sampling dates, and environmental metadata are provided in Supplementary Table S1.

**Figure 1:**
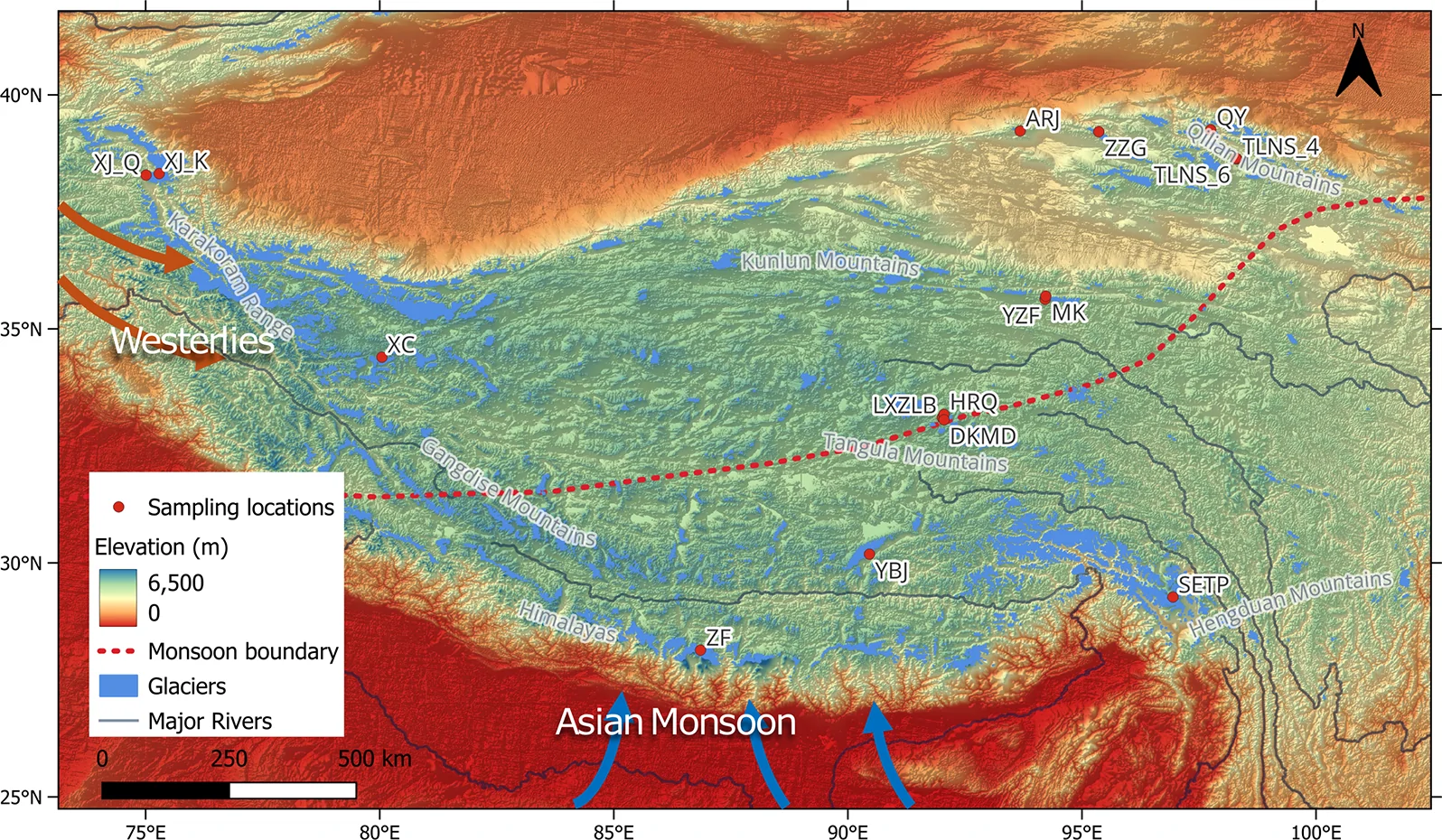
Sampling locations of glacier outflows across the Tibetan Plateau. Background colors show elevation, with shaded relief highlighting regional topography. Major mountain ranges and rivers are labeled to provide geographic context. Glacier outlines were obtained from the Second Glacier Inventory of China,^29^ and the monsoon boundary is based on Chen et al. (2019).^30^ Elevation data are from the USGS GTOPO30 digital elevation model. Hillshade and figure layout were generated in QGIS (v3.40.14).

In total, 30 samples were obtained, comprising a mid-ablation spatial data set and three glacier-specific time series. The spatial dataset consisted of 16 outflow samples collected during June–July, when meltwater flux was high. Three of these sites, Xiongcai: XC, Zhufeng: ZF, and southeastern Plateau: SETP, were also sampled repeatedly at approximately 20-day to monthly intervals throughout or within the ablation season to capture seasonal evolution, yielding 5, 6, and 6 samples, respectively. One mid-ablation sample from each time-series site was also included in the spatial data set. The SETP time series was collected from a glacier-fed proglacial lake (∼1 km^2^) rather than directly from an outflow stream.

At each site, samples were taken during local midday–afternoon, close to peak diurnal flow. Meltwater was collected into precleaned 20 L polyethylene canisters, with 40 L collected per sample to obtain sufficient DOM for solid-phase extraction and radiocarbon analysis. Separate subsamples for water chemistry were transferred to precleaned polycarbonate bottles. All samples were filtered through precombusted glass fiber filters (Whatman GF/F, 0.7 μm; 550 °C, 6 h). Filtrates were used for DOC quantification and DOM solid-phase extraction.

### 2.2 DOC Quantification, δ^18^O Isotope, and Anion Analyses

Dissolved organic carbon (DOC) was quantified as nonpurgeable organic carbon (NPOC) using a Shimadzu TOC-L CPH/CPN analyzer. Filtered samples were acidified in the instrument and purged to remove inorganic carbon before detection. A multipoint calibration was verified daily with check standards. Milli-Q water blanks were analyzed daily to determine instrument background, which was typically <0.1 mg C L^–1^ and was subtracted from sample signals. Samples were analyzed in duplicate, and duplicate results with a coefficient of variation <5% were accepted and averaged.

Water isotope (δ^18^O) values and nitrate (NO_3_^–^) and sulfate (SO_4_^2–^) concentrations were measured at the Key Laboratory of Tibetan Environment Changes and Land Surface Processes, Institute of Tibetan Plateau Research, Chinese Academy of Sciences (ITP-CAS), Beijing, China. δ^18^O was determined using a Picarro L2130-i cavity ring-down spectrometer, and the analytical precision was better than 0.1‰ for δ^18^O.^31^ NO_3_^–^ and SO_4_^2–^ concentrations were quantified by ion chromatography using a Thermo Scientific Dionex ICS-900 system equipped with a detection limit of 0.01 mg L^–1^.^32^

### 2.3 DOM Extraction and HR-MS Analysis

DOM was isolated by solid-phase extraction (SPE) following Dittmar et al. using styrene–divinylbenzene polymer cartridges (Bond Elut PPL, 500 mg, 6 mL; Agilent).^100^ Filtered samples were acidified to pH 2 with 12 M HCl, and loading volumes were adjusted based on measured DOC to target a carbon load of 2 mg DOC per cartridge. Cartridges were conditioned before extraction, rinsed after sample loading with pH 2 Milli-Q water to remove salts, dried under N_2_, and eluted with methanol (VWR Chemicals, HPLC-grade). SPE-DOC concentrations were quantified on a TOC analyzer (vario TOC cube, Elementar),^33^ and extracts were diluted to 20 mg C L^–1^ in 1:1 (v/v) methanol:Milli-Q water for mass spectrometric analysis.

HR-MS measurements were conducted on an Orbitrap Elite (Thermo Scientific) using direct infusion electrospray ionization in negative mode (ESI) following established protocols.^33,34^ Samples (100 μL) were infused at 20 μL min^–1^ in a 1:1 (v/v) methanol:Milli-Q mixture. The spray voltage was 2.65 kV. Mass spectra were collected over *m*/*z* 100–1000 at a nominal resolving power of 480,000 (at *m*/*z* 200). For each sample, at least 100 scans were averaged in Xcalibur (v3.0.63).

Raw Orbitrap files were converted to mzML using ThermoRawFileParser.^101^ Spectra were processed in R (v4.4.1) with MFAssignR (v1.0.2)^35^ for internal recalibration, peak alignment, and molecular formula assignment. Peaks with signal-to-noise (S/N) ≥ 5 were retained for formula assignment and downstream analyses. Molecular formulas were assigned with elemental constraints C1–60, H4–120, O1–30, N0–4, S0–2, P0–1, and mass error <0.6 ppm. SPE-PPL extraction procedural and solvent blanks were processed and analyzed, and formulas accounting for up to 95% of cumulative blank intensity were removed from further analysis. Formulas containing isotopes and formulas with multiple suggestions were also removed. Intensities were sum-normalized within each sample for intersample comparison.

From the assigned formulas, we calculated elemental ratios (H/C, O/C, N/C, S/C), the modified aromaticity index (AI_mod_),^36,37^ and the nominal oxidation state of carbon (NOSC).^38^ Intensity-weighted bulk parameters (e.g., mean H/C, O/C, *m*/*z*, NOSC, AI_mod_) were computed per sample from relative intensities. Molecular class composition was summarized using an operational scheme based on van Krevelen regions and AI_mod_ thresholds.^39^ Formulas were assigned to amino sugar-like (O/C = 0.56–0.95; H/C = 1.62–2.35; N > 0), carbohydrate-like (O/C = 0.56–1.23; H/C = 1.53–2.20), lignin-like (O/C = 0.21–0.44; H/C = 0.86–1.34; N = 0), lipid-like (O/C = 0.01–0.35; H/C = 1.34–2.18), peptide-like (O/C = 0.17–0.48; H/C = 1.33–1.84; N > 0), tannin-like (O/C = 0.16–0.84; H/C = 0.70–1.01; N = 0), aromatics-like (0.50 < AI_mod_ ≤ 0.66), and condensed aromatics (AI_mod_ > 0.66). Some formulas may be assigned to multiple classes since some class boundaries overlap. Formulas not meeting any class criteria were labeled “unassigned.” Class contributions were quantified as the summed relative intensity per class.

Putative pathway associations were explored by linking assigned formulas to KEGG compound identifiers and pathways via KEGGREST^40,41^ using an established R workflow.^42^ For each sample, pathway “abundance” was calculated as the summed relative intensity of formulas mapping to compounds within that pathway. This analysis provides comparative patterns and does not quantify in situ metabolic rates.

### 2.4 Radiocarbon Analysis

Radiocarbon content of SPE-DOM extracts (Δ^14^C) was measured following established protocols.^34,42,43^ Aliquots of DOM extracts were evaporated to dryness in precombusted tin capsules (target ∼300 μg C per sample), combusted to CO_2_ in an elemental analyzer, and reduced to graphite. Graphite targets were analyzed on a MICADAS accelerator mass spectrometer (Ionplus, Dietikon, Switzerland) at the Max Planck Institute for Biogeochemistry (MPI-BGC), Jena, Germany. Measurements were bracketed with size-matched modern (oxalic acid II) and ^14^C-depleted standards.^44,45^ Δ^14^C values and conventional radiocarbon ages (yBP) were reported following standard conventions with δ^13^C-based fractionation correction as provided by the AMS system. Radiocarbon measurements were successful for 23 samples; seven samples failed analysis or contained insufficient carbon for measurement.

### 2.5 Meteorological Data and Atmospheric Footprint (STILT) Analysis

Meteorological variables were extracted from the Third Pole Meteorological Forcing Data set (TPMFD; 1979–2023).^46^ For each sampling location, monthly precipitation, air temperature, and surface shortwave radiation were retrieved for 2018–2022 and averaged to annual means to represent contemporary climatological conditions around the sampling period.

Air-mass transport patterns for the ZF and XC time-series sites were assessed using the Stochastic Time-Inverted Lagrangian Transport (STILT) model.^47^ Unlike simple backward trajectories, STILT simulates ensembles of air parcels transported backward in time while explicitly accounting for turbulent mixing and convection, thereby representing key atmospheric mixing processes. For each sampling site, 100 particles were released hourly and traced backward for 10 days. Receptors were defined at a height of 20 m above ground level. Simulations were driven by European Centre for Medium-Range Weather Forecasts Integrated Forecasting System (IFS) meteorological fields at 3 h temporal and 0.25° × 0.25° spatial resolution. Monthly footprint fields were calculated for June–August at XC and June–October at ZF, corresponding to the sampling periods. Because meteorological forcing was available from 1 June, June simulations started on 10 June. Monthly cumulative footprints were normalized by the number of simulated footprints to allow comparison among months. Due to the complex topography of the Himalayan region, footprint results are interpreted qualitatively to identify dominant transport patterns rather than precise source attribution.

### 2.6 Statistical Analyses

All statistical analyses and visualizations were conducted in R (v4.4.1; RStudio). Variability in DOM molecular composition was performed using principal coordinates analysis (PCoA) based on Bray–Curtis dissimilarities calculated from Hellinger-transformed formula-intensity profiles. Intensity-weighted bulk molecular indices (e.g., elemental ratios, AI_mod_, NOSC, and *m*/*z*) and environmental variables were fitted as vectors onto the ordination using permutation-based fitting. To evaluate correlated spatial predictors, we used separate single-predictor PERMANOVA models based on Bray–Curtis dissimilarities, variation partitioning and partial distance-based redundancy analysis to quantify the unique and shared contributions of longitude, annual shortwave radiation, and annual precipitation. Adjusted *R* ^2^ values were reported, and significance was evaluated using permutation tests. Group differences in multivariate composition and dispersion were tested using PERMANOVA and betadisper, respectively. Group differences in DOM bulk parameters and molecular class contributions were evaluated using Kruskal–Wallis tests followed by Dunn’s post hoc comparisons with Benjamini–Hochberg adjustment. Associations between Δ^14^C and DOM composition, i.e., bulk indices and summed relative intensity of different molecular classes, were assessed with Spearman’s rank correlations for samples with Δ^14^C measurements (*n* = 23), followed by Benjamini–Hochberg correction. Because the Δ^14^C subset included seasonal observations from XC, ZF, and SETP, the reported correlations describe associations across the measured sample set rather than relationships among fully independent glacier systems. Parameter-level relationships were therefore additionally evaluated using rank-based mixed-effects models with glacier identity included as a random intercept. Formula-level correlations with Δ^14^C were calculated using relative intensities of formulas present in at least three samples, and formulas with |ρ| > 0.6 were retained as candidate radiocarbon-associated formulas.

## 3 Results

### 3.1 Spatial and Temporal Variation of DOC Concentration in Glacier Outflows

Dissolved organic carbon (DOC) concentrations in Tibetan Plateau glacier outflows were low overall but variable among sites and dates. Across all samples (*n* = 30), DOC ranged from 0.092 to 0.73 mg C L^–1^, with a median of 0.256 mg C L^–1^ (Table 1). These values fall within the range of previously reported DOC concentrations for Tibetan Plateau glacier-fed systems, which range from 0.16 to 4.94 mg C L^–1^, with a mean of 0.79 ± 0.68 mg C L^–1^.^48^ The highest DOC concentration was observed at ZF on 10 June, whereas the lowest was observed at the SETP glacier-fed proglacial lake on 11 September. DOC concentrations of the mid-ablation spatial data set exhibited a marginally negative correlation with longitude (*n* = 16; ρ = −0.47, *p* < 0.1). No consistent intraseasonal trend was evident within the XC, ZF, or SETP time series, likely reflecting short-term DOC variability driven by changing meteorological conditions, discharge regime, and hydrologic connectivity, as well as the limited temporal resolution of the available observations.^23^

**Table 1:** Overview of Sampling Dates, Site Information, and Environmental Characteristics of Glacier DOM Samples^a^

Name	Sampling time	Longitude	Annual sRAD (W m^–2^)	Annual precipitation (mm)	DOC (μg C L^–1^)	Δ^14^C-DOC (‰)	^14^C Age (yBP)	δ^18^O (‰)
Single-time samples								
XJ_K	2022–07–12	75.2888	214.1	339	226	–485.0	5331	–11.8
XJ_Q	2022–07–10	75.0088	214.4	128	373	–385.7	3914	–14.8
ARJ	2022–07–04	93.6807	188.2	344	258	–563.6	6661	–9.2
ZZG	2022–07–05	95.3601	190.2	406	140	–703.7	9771	–13.5
QY	2022–07–13	97.7519	191.8	513	218	–518.1	5864	–8.7
TLNS_4	2022–07–21	98.3068	195.2	604	232	–452.5	4839	–9.3
TLNS_6	2022–07–21	98.2930	195.4	583	311	–587.8	7119	–8.5
YZF	2022–06–29	94.2136	196.4	526	140	–512.6	5773	–8.8
MK	2022–07–10	94.2273	196.0	490	149	–517.8	5859	–9.2
LXZLB	2022–06–20	92.0159	217.4	618	370	N.A.	N.A.	–10.2
HRQ	2022–06–22	92.0536	216.7	577	209	N.A.	N.A.	–9.8
DKMD	2022–06–24	92.0518	217.6	617	150	–548.6	6389	–11.1
YBJ	2022–06–13	90.4610	207.2	792	255	–438.8	4640	–14.6
Time-series samples								
XC (*n* = 5)	2022–06–03 to 2022–08–10	80.0471	235.8	247	290–560	–463.2 to −323.7 (*n* = 3)	3142 to 4998	–14.6 to −10.7
ZF (*n* = 6)	2022–05–31 to 2022–10–10	86.8518	209.0	675	210–730	–782.5 to −349.7 (*n* = 6)	3457 to 12255	–20.4 to −18.8
SETP (*n* = 6)	2022–06–10 to 2022–11–01	96.9439	176.5	911	90–430	–659.4 to −509.5 (*n* = 3)	5722 to 8652	–16.4 to −14.1

a Annual sRAD denotes annual-mean shortwave radiation. Annual sRAD and precipitation values were derived from the Third Pole Meteorological Forcing Dataset (TPMFD; 1979–2023) as means over the period 2018–2022, see Section 2.5 for details. Values for time-series samples are reported as ranges across the sampling period; detailed values are provided in SI Table S1.

### 3.2 Regional Controls on Glacier Outflow DOM Composition among Sampled Tibetan Plateau Glaciers

Principal coordinates analysis (PCoA) of the spatial data set, including all mid-ablation samples across the Tibetan Plateau (*n* = 16), revealed DOM compositional variation associated with both molecular properties and regional environmental gradients (Figure 2). Despite low DOC concentration, glacier outflow DOM was generally enriched in lipid- and peptide-like formulas, consistent with previous observations of glacier-fed systems^5,9^ (Figure S1). However, substantial molecular heterogeneity occurred among samples (Figure 2).

**Figure 2:**
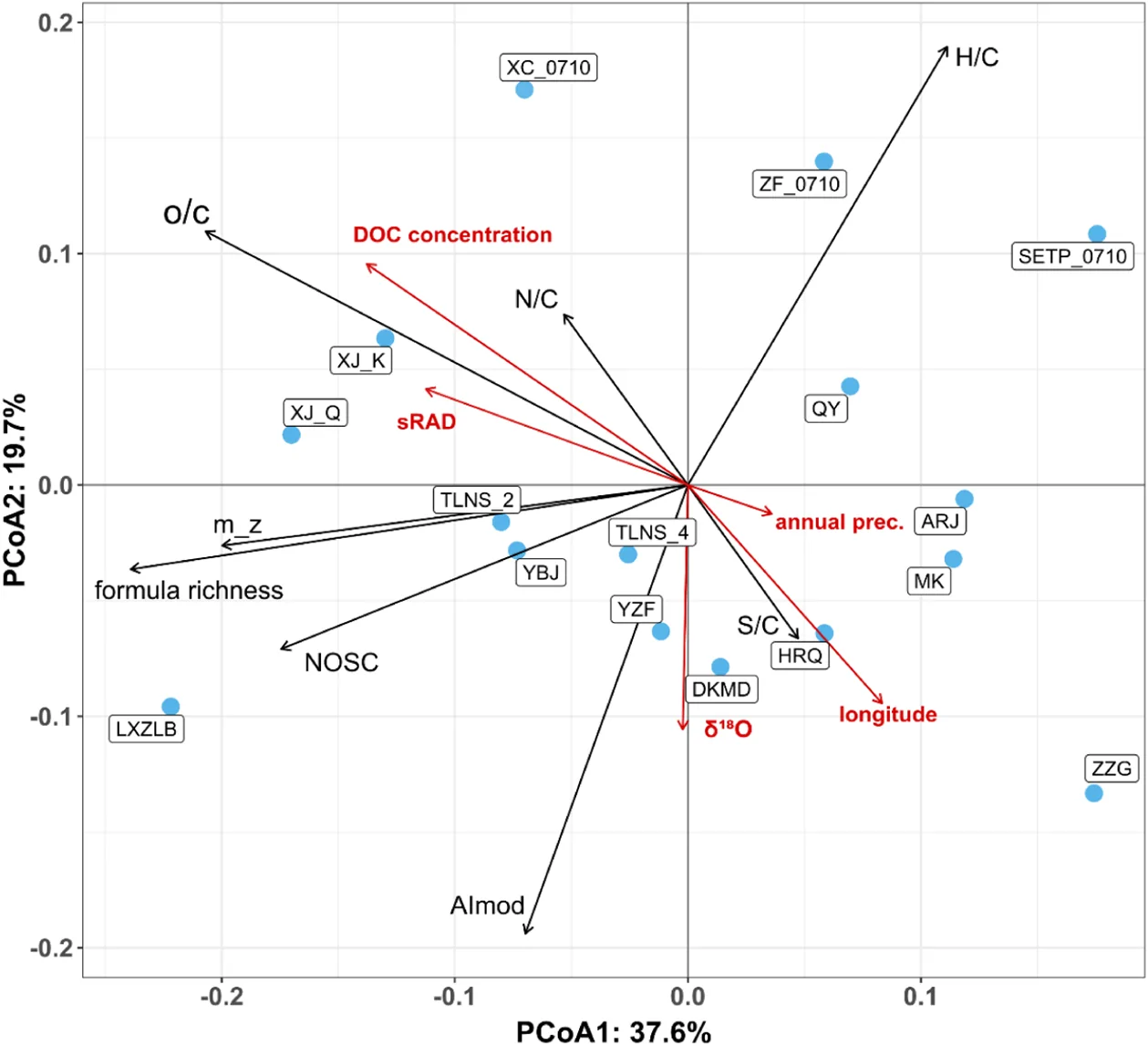
Principal coordinates analysis (PCoA) of glacier outflow DOM molecular composition across the Tibetan Plateau. Bray–Curtis dissimilarities were computed on Hellinger-transformed intensity profiles of assigned formulas. Vectors are fitted and represent formula richness (number of formulas) and intensity-weighted bulk parameters: NOSC (nominal oxidation state of carbon), m_z (molecular mass), AI_mod_ (modified aromaticity index), S/C, H/C, N/C, and O/C. Environmental factors include: longitude, annual precipitation, δ^18^O, and annual shortwave radiation.

The first two PCoA axes explained 37.6% (PCoA1) and 19.7% (PCoA2) of the variance, indicating pronounced differentiation among sites. Vectors of bulk molecular properties showed that formula richness, NOSC, and intensity-weighted *m*/*z* aligned strongly with PCoA1, suggesting that this axis mainly reflected gradients in molecular diversity, oxidation state, and molecular size.

Environmental fitting identified longitude and annual shortwave radiation as significant gradients associated with DOM compositional variability across the spatial data set, whereas δ^18^O and annual precipitation showed weaker associations (*p* < 0.1). In separate single-predictor PERMANOVA models, longitude and shortwave radiation were both significantly associated with DOM molecular composition, explaining 18.26% and 18.34% of the variation, respectively (both *p* < 0.01); no other available environmental variables showed significant associations (Table S3). However, variation partitioning showed that longitude, shortwave radiation, and annual precipitation jointly explained 13.7% of the adjusted compositional variation (*p* < 0.05), while none retained a significant unique contribution after accounting for the other predictors. The largest positive fraction was shared between longitude and radiation (Table S4). In the ordination, the high-radiation side of the gradient was associated with higher O/C and N/C.

Regional structure did not translate to uniformity among neighboring glaciers. Three samples (HRQ, DKMD, LXZLB) collected from different outlets of the same glacier complex (∼180 km^2^) showed marked compositional differences along PCoA1. LXZLB exhibited the highest formula richness (*n* = 5179), highest intensity-weighted molecular mass (296.69 Da), most oxidized DOM (NOSC = −0.33), and highest AI_mod_ (0.35) among the 16 spatial samples. In contrast, HRQ and DKMD had lower richness (3278 and 3151), lower NOSC (−0.52 and −0.48), and smaller molecular mass (255.42 and 261.46 Da), despite their geographic proximity (Table S2).

### 3.3 Seasonal Variation of DOM

#### 3.3.1 Overview of Seasonal Variability

The three time-series sites represent contrasting climatic settings across the Tibetan Plateau: XC is located in the arid, westerly-dominated northwestern Plateau with annual precipitation <250 mm; ZF is located on the northern slope of Mount Everest within the Westerly–South Asian monsoon transition zone, with 5-year mean annual precipitation of 675 ± 90 mm; and SETP is located in the humid southeastern Plateau under strong monsoon influence with 5-year mean annual precipitation of 911 ± 236 mm. Together, these sites capture a west-to-southeast climatic gradient for evaluating melt-season DOM dynamics across the Tibetan Plateau.

To evaluate seasonal variation relative to spatial heterogeneity, we compared three time-series sample sets with the mid-ablation spatial data set (Figure 3). Molecular class composition differed among the four groups (PERMANOVA on Bray–Curtis dissimilarities: *R* ^2^ = 0.34, *p* < 0.001), although within-group dispersion also varied (betadisper, *p* < 0.05), indicating that these differences reflected both shifts in group centroids and variation in within-group heterogeneity. The mid-ablation spatial data set itself showed broad compositional variability in relative intensities: lignin-like 12.08–24.73%, tannin-like 3.13–13.89%, aromatic-like 3.92–13.10%, condensed aromatics 1.90–8.58%, peptide-like 0.93–4.32%, and lipid-like 11.10–42.21%. Against this spatial background, seasonal variability differed strongly among the three time-series sites. ZF exhibited the greatest within-group dispersion, exceeding that of XC, SETP, and the spatial data set (Figure 3). This variability was especially pronounced for lignin-like (6.97–19.94%) and lipid-like (23.31–54.82%) classes, indicating strong intraseasonal compositional shifts. In contrast, XC and SETP showed more constrained variability across most classes. One exception was the peptide-like class at XC, which was both elevated and more variable than in other groups. The limited variability at SETP likely reflects its proglacial lake setting, where mixing, longer residence time, hydrological integration, and in-lake processing can buffer short-term changes in DOM composition.

**Figure 3:**
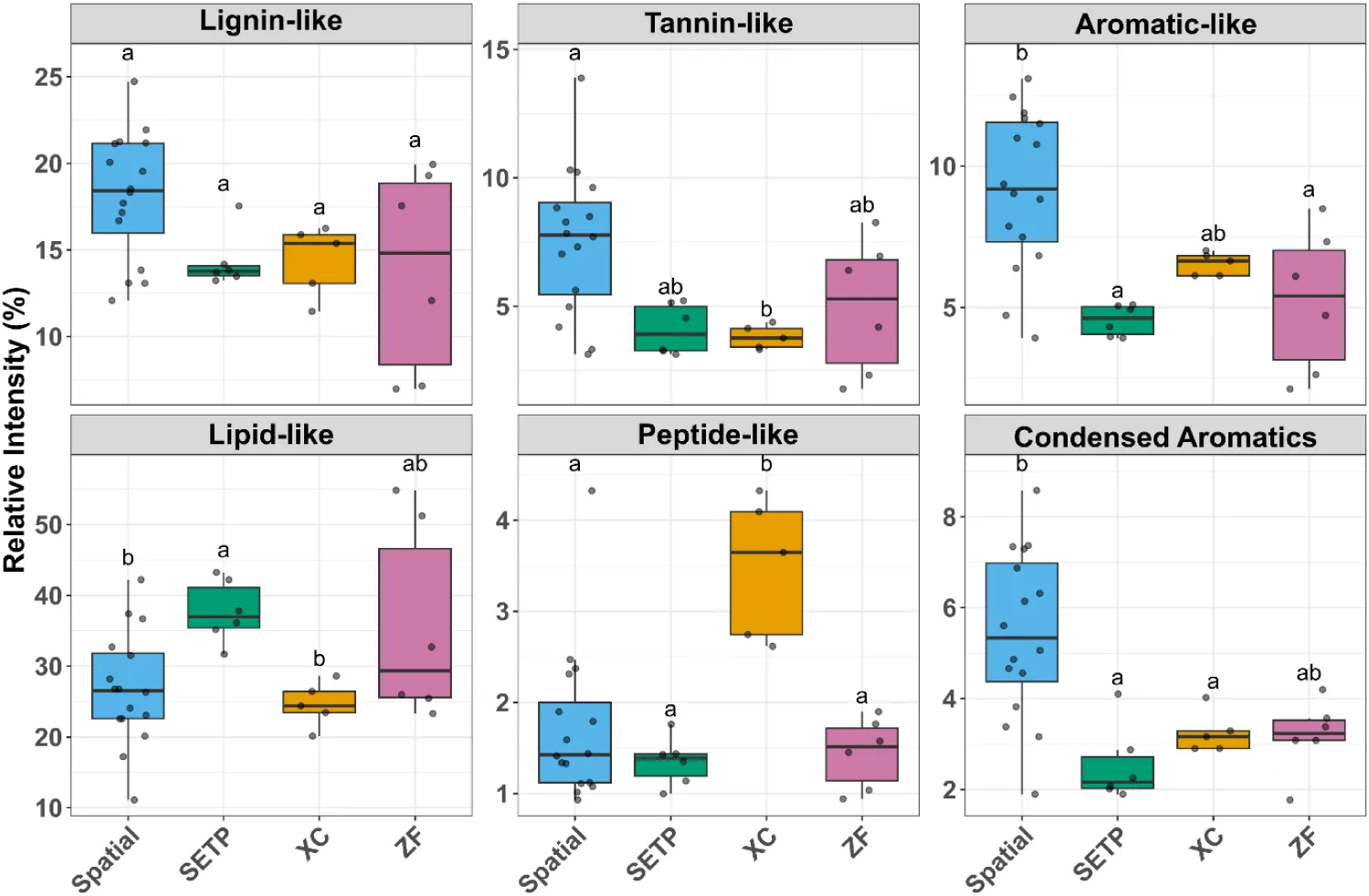
Relative intensity contributions of DOM molecular classes by sample set. Boxes show the median and interquartile range; whiskers extend to 1.5 × IQR; points are individual samples. Letters above boxes indicate pairwise differences among sample sets from Dunn’s tests with Benjamini–Hochberg FDR correction (α = 0.05); groups sharing a letter are not significantly different. The spatial data set comprises mid-ablation samples across multiple glaciers (including 10 July samples from XC, ZF, and SETP for comparability). SETP time-series samples were collected from a glacier-fed proglacial lake.

#### 3.3.2 Seasonal Evolution under Contrasting Dominant Controls

Because SETP samples were collected from a proglacial lake rather than a direct glacier outflow, the detailed seasonal comparison focuses on XC and ZF. These two glacier systems showed different seasonal DOM trajectories during the ablation period (Figure 4). XC exhibited constrained and largely nondirectional variability, whereas ZF showed a directional shift from lipid-like toward more aromatics and plant-associated molecular groups (i.e., lignin-, tannin-like compounds).

**Figure 4:**
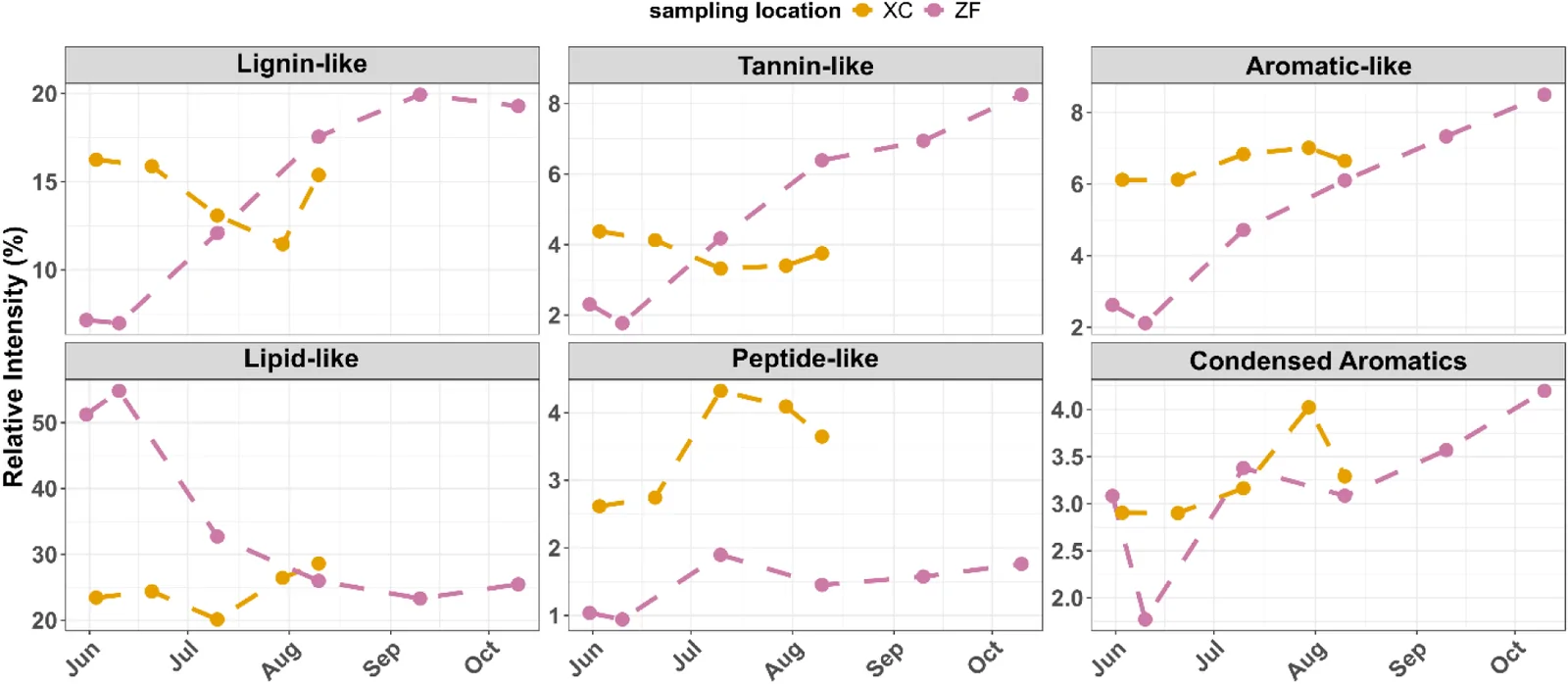
Seasonal trend of relative contributions of DOM molecular classes in XC and ZF time-series sample sets.

#### XC: Constrained Variability with Elevated Peptide-Like Signals

At XC, the relative intensity of lignin-like compounds decreased from 16.25% to 11.46% before returning to 15.38% in the final sample, while tannin-like compounds showed a similar but lower amplitude pattern (Figure 4). Aromatic-like contributions remained relatively stable (6.12–7.01%). In contrast, peptide-like signals were consistently elevated and dynamic, peaking at 4.32% before declining to 3.65%. Across the full data set, peptide-like contributions were positively correlated with shortwave radiation (Spearman ρ = 0.54, *p* < 0.05) (Figure S2). Consistent with this pattern, KEGG-based annotations indicated higher relative abundance of formulas associated with amino acid biosynthesis and carbon fixation pathways at XC (Figure 5a). STILT footprint fields aggregated over the sampling periods (Figure S3) indicate that the core region of elevated surface influence (>1 × 10^–4^ ppm μmol^–1^ m^2^ s) at XC was comparatively constrained and persistent from June to August, although broader low-to-moderate surface influence (<1 × 10^–4^ ppm μmol^–1^ m^2^ s) expanded transiently in August.

**Figure 5:**
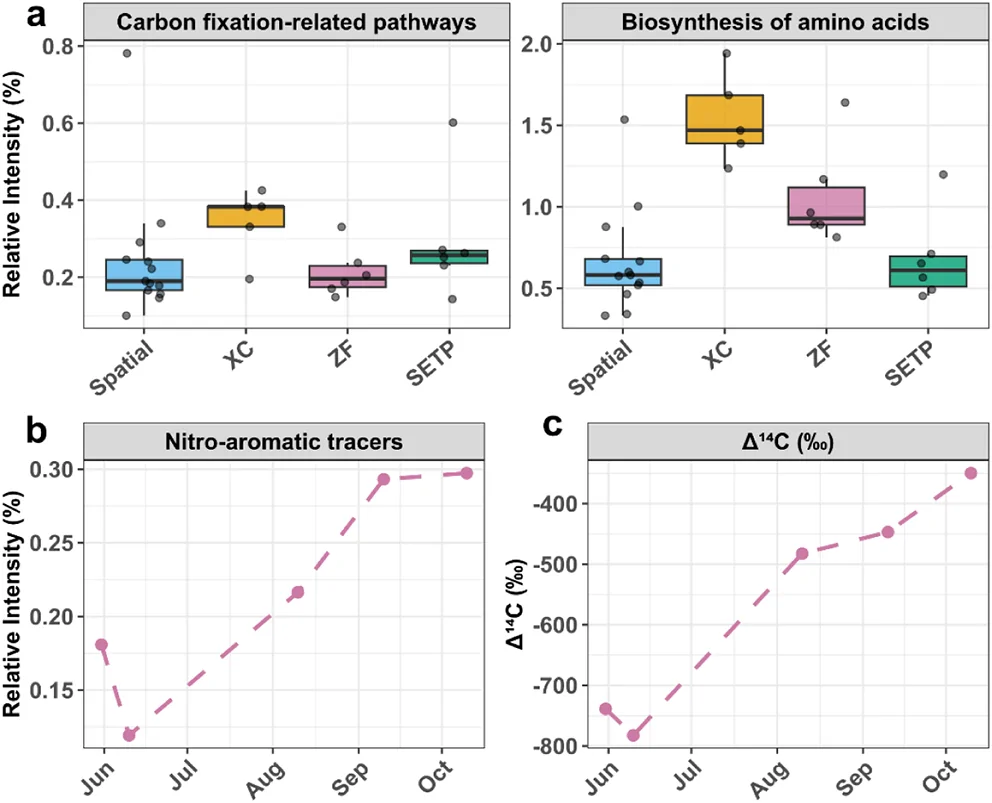
(a) Relative intensities of KEGG-predicted carbon fixation and amino acid biosynthesis pathways across spatial samples and glacier-specific time series (XC, ZF, and SETP). SETP samples were collected from a glacier-fed proglacial lake. (b) Seasonal variation in nitro-aromatic tracer intensity in ZF DOM. In (a) and (b), values represent the summed relative intensities of formulas assigned to each pathway or group. (c) Seasonal variation in Δ^14^C of SPE-DOM at ZF.

#### ZF: Directional Increase in Aromatic and Plant-Associated Classes

At ZF, lignin-like, tannin-like, and aromatic-like formulas increased progressively during the ablation season, indicating a shift toward more plant-associated and aromatic DOM. Relative intensities increased from 7.15% to 19.29% for lignin-like compounds, from 2.31% to 8.25% for tannin-like compounds, and from 2.62% to 8.49% for aromatic-like compounds, whereas lipid-like contributions decreased from 51.23% to 25.45% (Figure 4). Independent tracers further support increasing allochthonous influence. The relative intensity of 15 putative nitro-aromatic biomass-burning tracers increased from 0.18% to 0.29% (Figure 5b). Inorganic atmospheric tracers including NO_3_^–^ and SO_4_^2–^, also increased over the season (Table S1), and NO_3_^–^ correlated positively with terrestrial-associated classes (lignin-like, tannin-like, aromatics-like, condensed aromatics) but negatively correlated with amino sugar-like, carbohydrate-like, and lipid-like classes (Figure S2). STILT footprint maps (Figure S4) indicated a broad and seasonally variable footprint distribution at ZF, with continued surface influence from the Himalayan and southern Tibetan Plateau region during the ablation season. Although aromatic and condensed aromatics increased, Δ^14^C values of SPE-DOM became progressively less depleted, from −738.8‰ to −349.7‰ (Figure 5c), indicating increasing contributions of relatively modern rather than predominantly fossil carbon.

### 3.4 Molecular Composition and Radiocarbon Age

Radiocarbon (Δ^14^C) provides constraints on the apparent age and potential sources of exported DOM. SPE-DOM Δ^14^C values ranged from −782.5‰ to −323.7‰ across the 23 measured samples, corresponding to apparent conventional radiocarbon ages of approximately 3.1–12.3 kyr BP (Table 1). We related Δ^14^C of DOM to intensity-weighted bulk indices and molecular class contributions (Figure 6a). Δ^14^C was positively associated with formula richness and NOSC, intensity-weighted *m*/*z* and peptide-like contributions, indicating that more modern DOM tended to be more molecularly diverse, more oxidized, and more peptide-associated. The fraction of unassigned signal increased with Δ^14^C, suggesting that part of the modern-associated pool was not captured by the molecular class simply defined based on elemental ratios and AI_mod_. These principal relationships were retained in rank-based mixed-effects models that included glacier identity as a random intercept (Figure S6). In particular, lipid-like contribution showed a strong negative correlation with Δ^14^C (ρ = −0.73), and this association remained after accounting for glacier-level clustering (standardized slope = −0.72, 95% CI: −1.01 to −0.44, BH-adjusted *p* < 0.001), indicating that lipid-enriched DOM was associated with older radiocarbon signatures.

**Figure 6:**
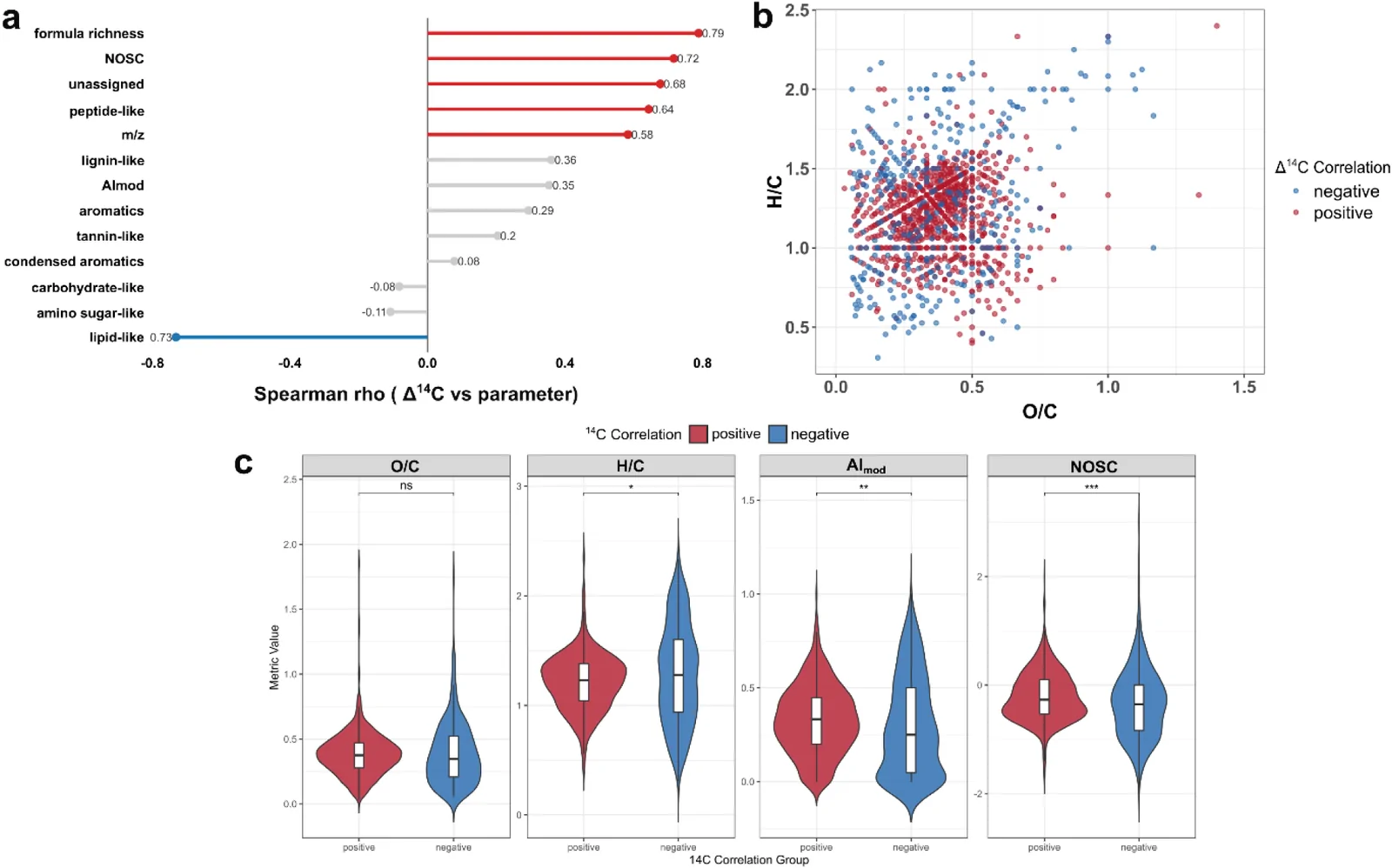
(a) Spearman correlations of Δ^14^C with bulk DOM indices and classes; (b) formulas whose relative intensity has either positive or negative correlation with Δ^14^C (criteria: |ρ| > 0.6, presence in ≥3 samples); (c) molecular indices of these two pools of formulas.

To examine composition-radiocarbon coupling at the formula level, we performed an exploratory screening of individual formulas associated with Δ^14^C (|ρ| > 0.6, presence in ≥3 samples). This yielded 877 formulas positively correlated with Δ^14^C (“modern-associated”) and 367 negatively correlated (“old-associated”) (Figure 6b). In van Krevelen space, the old-associated formulas were broadly distributed and included many high-H/C features (H/C > 1.5), consistent with the class-level negative relationship between lipid-like contributions and Δ^14^C. The modern-associated formulas were more concentrated, with many formulas at H/C < 1.5 and O/C < 0.5. Molecular indices distinguished these two formula pools (Figure 6c). Compared with modern-associated formulas, old-associated formulas had a relatively higher H/C, lower AI_mod_, and lower NOSC, indicative of a more reduced composition. Notably, the old-associated pool also contained a subset of highly condensed aromatic formulas (AI_mod_ > 0.66), demonstrating that the old-associated DOM pool was chemically heterogeneous, comprising both reduced lipid-like formulas and condensed aromatics. It should be noted that the formula-level correlations represent exploratory associations across the measured samples and should not be interpreted as independent among-glacier relationships.

## 4 Discussion

### 4.1 Regional Climatic Controls on DOM Molecular Composition

The main regional gradient in DOM composition across the sampled spatial data set was aligned with longitude and shortwave radiation (Figure 2), and both variables were significant in single-predictor PERMANOVA models (Table S3). Variation partitioning showed that much of their explanatory power was shared (Table S4), suggesting that they captured overlapping components of a broader regional environmental gradient and that longitude primarily acted as a geographic proxy for this transition. Shortwave radiation is spatially heterogeneous across the Tibetan Plateau rather than following a simple monotonic east–west pattern,^21,49^ but during the main ablation period represented by our sampling campaign, the western and northwestern sites in our data set generally experienced higher shortwave radiation than the eastern and southeastern sites (Figure S5). The observed compositional gradient is therefore broadly consistent with a transition from wetter, monsoon-influenced conditions in the southeast and east to drier, more strongly westerly influenced, and higher-radiation conditions in the west and northwest. Previous studies have shown that this regional climatic gradient also affects DOC concentrations in glacier-related systems, including glacier runoff, rivers, and snow pits, with generally higher DOC concentrations in northwestern regions linked to stronger mineral dust input and evaporation, and lower DOC concentrations in southeastern regions linked to stronger precipitation dilution.^15,20,48^ Similar regional contrasts have also been reported for DOM compositions in Tibetan Plateau cryoconite environments, where monsoon-region glaciers showed higher relative intensities of carbohydrate-, lignin-, tannin-, and condensed aromatic-like compounds than central Plateau glaciers, likely reflecting stronger terrestrial biomass and anthropogenic inputs.^28^

In our data set, the high-radiation side of the spatial gradient was associated with higher O/C and N/C, suggesting that radiation-linked photochemical and biological processes may contribute to regional differences in glacier outflow DOM composition. Previous studies have shown that photochemical processing can strongly alter DOM molecular composition in aquatic and cryospheric environments.^17,18^ Photodegradation can preferentially transform aromatic and high-molecular-weight compounds, enrich the remaining molecular pool in N-containing formulas,^17^ increase O/C through oxidative modification of aromatic structures,^12^ and generate more bioavailable substrates for microbial utilization.^17,18,50^ These mechanisms provide a plausible explanation for the higher O/C and N/C observed toward the high-radiation side of the spatial gradient. At the same time, glacier-associated microbial communities, especially phototrophic microorganisms in supraglacial environments, can produce labile lipid- and peptide-like compounds through biomass production and exudation.^51,52^ Therefore, the molecular patterns observed in the arid, high-radiation region likely reflect coupled photochemical transformation and microbial processing.

In contrast, DOM signatures in monsoon-influenced or mixed-circulation catchments may be shaped by more variable atmospheric inputs. Precipitation and air-mass transport across the Tibetan Plateau can deliver complex mixtures of mineral dust, biogenic organic matter, and anthropogenic aerosols.^28^ Such inputs may superimpose diverse allochthonous sources on local photochemical and microbial processing, explaining why eastern and monsoon-affected samples were not characterized by a single, uniform molecular signature.

Despite these plateau-scale gradients, neighboring outlets draining the same glacier complex differed markedly in DOM composition (i.e., LXZLB, HRQ, DKMD) (Table S2), particularly along the primary ordination axis associated with formula richness, NOSC, and intensity-weighted *m*/*z*. This result highlights the importance of local-scale controls that can overlay regional climatic forcing, including glacier aspect and shading, debris cover and cryoconite development, subglacial routing and residence times, and proglacial soil and vegetation development.^4,14,53,54^ Such small-scale heterogeneity is consistent with previous glacier studies showing that proglacial outflow DOM can differ substantially from supraglacial DOM because downstream transport integrates surface-derived material, subglacial inputs, and catchment-derived organic matter.^55,56^ In this context, adjacent glacier outlets may export chemically distinct DOM even under similar regional climate conditions, depending on their hydrological pathways and degree of interaction with glacier surfaces, sediments, and proglacial terrain.

Taken together, our results suggest that the broad geographic and climate gradients provide an important control on glacier outflow DOM composition among the sampled Tibetan Plateau glaciers. However, because the 16 sites were unevenly distributed, with limited coverage in the western Plateau, the identified gradient should not be considered spatially representative of the entire region. Local hydrologic connectivity and catchment context also generate substantial variation among neighboring outlets, limiting predictions at the individual-glacier scale.

### 4.2 Seasonal Evolution under Contrasting Dominant Controls

The contrasting seasonal trajectories at XC and ZF show that melt-season DOM dynamics did not follow the same seasonal pattern across the Tibetan Plateau. XC showed relatively constrained seasonal variability with persistently high peptide-like contributions, whereas ZF showed a directional shift toward aromatic and plant-associated molecular classes. These patterns suggest that seasonal DOM evolution depends on the balance among internal glacier processing, changing meltwater connectivity, and external atmospheric or terrestrial inputs.

#### XC: Constrained Seasonal Shifts with an Enhanced Autochthonous Imprint

At XC, peptide-like compounds showed higher relative contributions than in other sample sets, with a mean relative intensity of 3.5% (Figure 4 and Table S2), exceeding the mean value reported for a global glacier outflow data set (2%, *n* = 45).^9^ This pattern, together with higher relative abundances of formulas associated with KEGG-based amino acid biosynthesis and carbon fixation pathways (Figure 5a), suggests a stronger autochthonous molecular signature within the glacier system. As discussed above, XC is located in an arid, high-radiation setting where photochemical transformation and microbial production may both be important. The comparatively constrained STILT footprints during the sampling period at XC also suggest that the external atmospheric transport was less variable at XC than at ZF (Figures S3 and S4), which may explain why terrestrial- and aromatic-associated molecular classes did not show the same coherent seasonal enrichment as observed at ZF.

High eolian mineral dust deposition in the westerly-dominated region may also enhance microbial activity and in situ organic matter production by facilitating cryoconite development on glacier surfaces.^20^ Cryoconite environments are microbial hotspots and commonly contain abundant CHO- and CHON-containing formulas.^4,14,16^ A study of cryoconite from northern and central Tibetan Plateau glaciers found that N-containing formulas accounted for about 38% of identified molecules, many with relatively high N/C ratios.^16^ These characteristics are consistent with the elevated peptide-like and N-containing signals observed at XC, and support the interpretation that internal glacier-surface microbial processes contributed to DOM export at this site.

Seasonal trajectory in peptide-like DOM at XC may also reflect evolving hydrological connectivity during the melt season. Early-season runoff is often dominated by distributed subglacial drainage that exports basal and sediment-associated organic matter, which can be relatively aromatic and terrestrially influenced.^22^ As the season progresses and channelized drainage develops, supraglacial inputs and freshly produced microbial DOM can be flushed more efficiently into outflow waters, increasing protein- and peptide-like signatures.^5,9,57^ The observed oscillations in peptide contributions at XC (Figure 4) are therefore consistent with a system in which internal processing and evolving drainage connectivity modulate DOM composition, producing seasonal variability without a clear directional shift.

#### ZF: Directional Seasonal Shifts Consistent with Increasing Atmospheric Influence

In contrast to XC, ZF exhibited increasing contributions of aromatic-like and plant-associated molecular classes, accompanied by increases in nitro-aromatic biomass-burning tracers and inorganic atmospheric tracers. Together with the broad and seasonally variable STILT footprints, including sustained atmospheric connectivity to the Himalayan and southern Tibetan Plateau region, these patterns indicate a seasonally strengthening allochthonous imprint at ZF. This imprint may reflect enhanced atmospheric deposition, seasonal changes in source strength, and progressive meltwater-mediated release of terrestrially associated material deposited on glacier surfaces or mobilized from the glacier forefield during the ablation season.^19,58,59^

Such an interpretation fits the broader atmospheric setting of the southern Tibetan Plateau, which is frequently influenced by South Asian air masses transporting mixtures of anthropogenic and biogenic aerosols, including biomass-burning products and fossil fuel emissions.^19,28,58–60^ These inputs can contribute aromatic and plant-associated signals to glacier surfaces and subsequently to outflow waters, particularly during the monsoon and ablation season.^15^ High aromaticity and abundant condensed aromatic compounds have also been reported in many southern Tibetan Plateau glacier-related environments, including cryoconite, snow pits, precipitation, and rivers.^11,28^ For example, one river study reported that condensed aromatics accounted for 31–39% of assigned molecular formulas, largely attributed to anthropogenic input.^11^

Importantly, Δ^14^C of SPE-DOM at ZF became less depleted as aromatic contributions increased with the season (Figure 5c), indicating that the increasing aromatic pool was not dominated by radiocarbon-dead fossil sources. Instead, this pattern is more consistent with inputs of relatively recent carbon, including modern terrestrial material and biomass-burning products. This interpretation is supported by one radiocarbon-based assessment suggesting that ∼80% of DOC in snow deposition in the Tibetan Plateau is nonfossil in origin during both summer and winter.^60^ In addition, another study also found that approximately half of the black carbon deposited on Himalayan glaciers originates from biomass combustion.^58^

Overall, these different trajectories indicate that melt-season DOM evolution in Tibetan glacier outflows is strongly site-dependent: internal microbial and photochemical processing appear to dominate at XC, whereas atmospheric and terrestrial inputs become increasingly important at ZF.

### 4.3 Composition–Radiocarbon Coupling and Sources of Aged Carbon

The strongly depleted Δ^14^C values (−782.5‰ to −323.7‰, i.e., 12.3–3.1 kyr BP) indicate that Tibetan glacier outflows exported substantially preaged DOM. The oldest values in our data set were more depleted than previously reported for Tibetan Plateau glacier surface ice in Mount Nyainqentanglha (−158.3‰), and glacier-stream DOC from the Karola Glacier stream (−259.2‰),^10^ and corresponded to apparent radiocarbon ages older than the reported ^14^C age of the East Rongbuk ice core from the same sampling region (6.75 kyr).^61^ This suggests that some of the outflows sampled here contained a particularly aged DOM component.^10,19^ Similarly depleted or even older carbon has been observed in other glacierized systems, including snow from the Juneau Icefield in Alaska (−743‰)^62^ and glacier outflows in southeast Alaska (−810.1‰ to −896.9‰),^25^ where aged carbon has been linked to anthropogenic fossil-fuel combustion aerosol inputs. Highly radiocarbon-depleted organic carbon has also been reported in subglacial sediments from Arctic glaciers, with Δ^14^C values approaching −1000‰,^63^ indicating that subglacial sedimentary reservoirs can contain very old carbon. Together, these comparisons show that highly aged carbon in glacierized systems may derive from multiple potential reservoirs, including atmospheric deposition, snow and ice archives, and subglacial sediments. The composition–radiocarbon relationships in our data set showed lower Δ^14^C values were most strongly associated with lipid-like and high-H/C formulas, rather than with aromatic or conventionally recalcitrant molecular signatures. This pattern highlights a disconnect between radiocarbon age and common molecular indicators of reactivity in our data set, suggesting that aged glacier DOM is not necessarily dominated by highly aromatic or chemically recalcitrant structures. We note that lipid-like and high-H/C formulas are interpreted here as indicators of potential lability based on molecular composition, rather than as direct measurements of bioavailability.

Several mechanisms could produce such an “aged lipid-like” signal. One plausible contributor is petrogenic or sediment-associated organic matter mobilized by subglacial erosion and ice-rock interactions. Although petrogenic organic carbon is often considered relatively recalcitrant and low in organic carbon content, dissolved organic fractions released from carbonate rock powders have been reported to include substantial lipid-, peptide-, and carbohydrate-like compounds, collectively accounting for 39.2% and 28% of total DOM in two different carbonate rock samples, and to be readily consumed during microbial incubations.^64^ This suggests that rock-derived or sediment-associated DOM can contain chemically reduced and potentially bioavailable fractions, rather than only highly condensed or recalcitrant material.

A second possible contributor is subglacial microbial production and turnover supported by ancient inorganic or organic carbon sources.^53,65^ Subglacial environments can sustain microbial communities at the sediment–bedrock interface, where physical grinding and mineral weathering release reduced compounds and inorganic carbon that can support chemolithoautotrophic metabolism.^66,67^ If these microbial communities use radiocarbon-depleted DIC, fossil organic matter, or old sedimentary substrates, subsequent biomass production, necromass accumulation, and microbial reworking could contribute lipid- and peptide-like compounds with depleted Δ^14^C signatures to the DOM pool.^63,68^ The seasonal pattern at ZF supports the presence of the aged lipid-like DOM component: the two earliest samples had both the lowest Δ^14^C values (−738.8‰ and −782.5‰) and the highest lipid-like contributions (51.2% and 54.8%), consistent with a stronger basal or subglacial contribution to early-season runoff and greater interaction with sediments and bedrock.^63^

Aromatic and condensed aromatic-like formulas showed a different relationship with radiocarbon content. Unlike lipid-like formulas, they did not display a clear negative association with Δ^14^C across our data set (Figure 6a). This differs from the global glacier data set of Holt et al. (2024),^8^ in which the aged DOM pool was more aromatic and was interpreted to reflect fossil-fuel combustion-derived inputs. In Tibetan Plateau glacier outflows, however, aromatic and condensed aromatic-like formulas may integrate both fossil and nonfossil sources. As discussed above, previous studies from the Tibetan Plateau and Himalayan region indicate substantial nonfossil carbon in snow deposition and an important contribution of biomass combustion to glacier-deposited black carbon.^19,58,60^ Because the relative contributions of these external inputs vary across regions and seasons, their net radiocarbon signal may not align predictably with carbon age.

Overall, the composition–radiocarbon coupling observed here indicates that glacier outflows on the Tibetan Plateau export a complex mixture of modern, peptide-associated DOM and aged DOM that includes chemically reduced lipid-like formulas. Resolving the origin of the “old-but-potentially labile” component will require targeted characterization of subglacial microbial communities and better constraints on bedrock and sediment organic carbon sources beneath Tibetan Plateau glaciers.

### 4.4 Synthesis and Implications

Glacier outflow DOM on the Tibetan Plateau was heterogeneous across space and dynamic through the melt season, reflecting the combined influence of regional climatic setting, meltwater routing, atmospheric inputs, and local catchment processes. In the spatial data set, longitude and shortwave radiation captured a broad east–west geographic–climatic gradient that covaried with DOM molecular composition, whereas the seasonal data sets revealed contrasting DOM trajectories among glacier environments. At the monsoon-influenced ZF glacier, increasing aromatic and plant-associated molecular signatures, together with more modern Δ^14^C values, were consistent with greater contributions of recent external or near-catchment carbon during the melt season. In contrast, at the arid, high-radiation XC glacier, more constrained DOM variability and elevated peptide-like signatures suggested a stronger role of in-glacier or near-glacier processing, including microbial activity and photochemical alteration. Together, these patterns show that glacier outflows are not passive conduits of a uniform glacial DOM pool, but active biogeochemical interfaces where source mixing and transformation shape the DOM exported downstream.

A key finding, but also a central unresolved question, is the origin and ecological significance of the old but potentially labile DOM pool. Distinguishing among its possible sources will require a source-explicit sampling strategy that directly links glacier outflow DOM to candidate materials and hydrological pathways in the surrounding glacier system. Future studies could compare outflow DOM with DOM leached from locally relevant endmembers, particularly bedrock, sediments, and soils collected near glacier outlets and within proglacial areas.^63,69–71^ Combining these endmember comparisons with HR-MS and bulk, fraction-specific, or compound-specific δ^13^C and Δ^14^C measurements would provide stronger constraints on the origin of the old high-H/C fraction. In parallel, biodegradation incubations coupled with HR-MS and radiocarbon measurements would help determine whether this fraction is actively consumed or transformed, or is largely transported downstream without substantial alteration.

By resolving both the origin and reactivity of old high-H/C DOM, future studies can better predict how glacier retreat will alter carbon quality, microbial energy supply, and ecosystem functioning in high-mountain headwaters.

## Supplementary Material



## Notes

Additional information regarding sampling information, environmental variables, and chemical and isotopic parameters for all samples (Table S1); molecular composition and compound-class contributions of DOM (Table S2); exploratory analyses of associations between various variables (continuous environmental variables and categorical site characteristics) and DOM molecular composition in the spatial data set (Table S3); variation partitioning results (Table S4); and additional figures showing DOM composition across water types, relationships with environmental variables, STILT footprint fields indicating potential upwind source regions, spatial distribution of surface downwelling shortwave radiation across the Tibetan Plateau, and associations between Δ^14^C and DOM molecular parameters evaluated using rank-based linear mixed-effects models with glacier identity as a random intercept (Figures S1–S6) (PDF)
