# Supplementary material for: Air-Mass Influence, Radiation, and Melt-Season Dynamics Jointly Shape Dissolved Organic Matter Composition and Radiocarbon Age in Glacier Outflows on the Tibetan Plateau
Source: Environ Sci Technol. 2026 Aug 5;60(32):22578–90. doi: 10.1021/acs.est.6c06257 (PMC13492468; doi:10.1021/acs.est.6c06257)
Supplement: Supplementary file 1 [file es6c06257_si_001.pdf]

Supporting Information for

Air-mass influence, radiation, and melt-season dynamics jointly shape dissolved organic matter composition and radiocarbon age in glacier outflows on the Tibetan Plateau

Chen Huang [a], Mo Wang [b]*, Xiaolong Zhang [b], Frank-Thomas Koch [c], Christoph Gerbig [c], Hongxing Jiang [d], Baiqing Xu [b], Gerd Gleixner [a]*

[a] Department of Biogeochemical Processes, Max Planck Institute for Biogeochemistry, Jena, 07745, Germany

[b] State Key Laboratory of Tibetan Plateau Earth System, Environment and Resources, Institute of Tibetan Plateau Research, Chinese Academy of Sciences, Beijing, 100101, China

[c] Department of Biogeochemical Signals, Max Planck Institute for Biogeochemistry, Jena, 07745, Germany

[d] Shanghai Key Laboratory of Air Quality and Environmental Health, Department of Environmental Science and Engineering, Fudan University, Shanghai, 200433, China

*Corresponding authors:

Mo Wang (wangmo@itpcas.ac.cn); Gerd Gleixner (gerd.gleixner@bgc-jena.mpg.de)

This PDF includes:

Number of pages: 16

Number of tables: 4

Number of figures: 6

Table S1. Sampling information, environmental variables, and measured chemical and isotopic parameters for all glacier outflow samples.

Full name	Abbr.	Sampling time	Latitude	Longitude	Elevation (m)	Annual SRAD (W m^{2-})	Annual precipitation (mm)	DOC (mg C L^{-1})	NO_3^- (mg L^{-1})	SO_4^{2-} (mg L^{-1})	$\Delta^{14}C$-DOC (‰)	$\delta^{18}O$ (‰)
Single sampling												
Xinjiang_Kuokesayi	XJ_K	2022-07-12	38.3181	75.2888	3915	214.11	339	0.2259	1.00	8.34	-485	-11.77
Xinjiang_Qiaodumake	XJ_Q	2022-07-10	38.2859	75.0088	4219	214.40	128	0.3725	0.78	3.25	-385.7	-14.81
Aerjin	ARJ	2022-07-04	39.2277	93.6807	4722	188.17	344	0.2579	5.97	92.80	-563.6	-9.24
Zhazigou	ZZG	2022-07-05	39.2119	95.3601	4302	190.23	406	0.14	0.55	0.79	-703.7	-13.51
Qiyi	QY	2022-07-13	39.2529	97.7519	4304	191.83	513	0.2184	1.02	47.52	-518.1	-8.70
Tuolenanshan	TLNS_4	2022-07-21	38.6329	98.3068	4453	195.15	604	0.232	2.99	29.98	-452.5	-9.32
Tuolenanshan	TLNS_6	2022-07-21	38.6452	98.2930	4578	195.35	583	0.311	2.03	32.07	-587.8	-8.49
Yuzhufeng	YZF	2022-06-29	35.6321	94.2136	5057	196.43	526	0.1396	1.76	5.37	-512.6	-8.77
Meikuang	MK	2022-07-10	35.6998	94.2273	4462	195.95	490	0.1486	3.60	88.62	-517.8	-9.16
Longxiazalongba	LXZLB	2022-06-20	33.1046	92.0159	5204	217.35	618	0.3697	1.77	20.38	N.A.	-10.22
Hariqin	HRQ	2022-06-22	33.1736	92.0536	5153	216.73	577	0.2092	1.05	11.23	N.A.	-9.82
Dongkemadi	DKMD	2022-06-24	33.0556	92.0518	5253	217.58	617	0.1497	1.10	1.55	-548.6	-11.06
Yangbajing	YBJ	2022-06-13	30.1865	90.4610	5487	207.23	792	0.2549	0.57	6.08	-438.8	-14.58
time-series sampling												
Xiongcai	XC_0603	2022-06-03	34.3944	80.0471	5381	235.78	247	0.4023	2.80	5.60	-463.2	-12.36
	XC_0620	2022-06-20	34.3944	80.0471	5381	235.78	247	0.4758	3.35	6.47	N.A.	-10.73
	XC_0710	2022-07-10	34.3944	80.0471	5381	235.78	247	0.2857	0.35	1.32	N.A.	-13.74

Full name	Abbr.	Sampling time	Latitude	Longitude	Elevation (m)	Annual SRAD (W m^{2-})	Annual precipitation (mm)	DOC (mg C L^{-1})	NO_3^- (mg L^{-1})	SO_4^{2-} (mg L^{-1})	$\Delta^{14}C$-DOC (‰)	$\delta^{18}O$ (‰)
	XC_0730	2022-07-30	34.3944	80.0471	5381	235.78	247	0.5588	1.61	3.62	-323.7	-12.67
	XC_0810	2022-08-10	34.3944	80.0471	5381	235.78	247	0.2991	0.97	3.04	-394.7	-14.58
Zhufeng	ZF_0531	2022-05-31	28.1346	86.8518	5154	208.95	675	0.5375	0.00	10.07	-738.8	-19.66
	ZF_0610	2022-06-10	28.1346	86.8518	5154	208.95	675	0.7345	0.00	7.81	-782.5	-19.37
	ZF_0710	2022-07-10	28.1346	86.8518	5154	208.95	675	0.3993	0.06	7.11	-698.6	-18.81
	ZF_0810	2022-08-10	28.1346	86.8518	5154	208.95	675	0.3378	0.12	15.96	-482.6	-20.39
	ZF_0910	2022-09-10	28.1346	86.8518	5154	208.95	675	0.2070	0.11	16.51	-447	-19.89
	ZF_1010	2022-10-10	28.1346	86.8518	5154	208.95	675	0.3612	0.15	25.82	-349.7	-20.15
Southeast Tibaten Plateau_Palong	SETP_0610	2022-06-10	29.2701	96.9439	4641	176.50	911	0.1027	0.30	34.07	-509.5	-14.09
	SETP_0710	2022-07-10	29.2701	96.9439	4641	176.50	911	0.1191	0.13	33.68	N.A.	-14.65
	SETP_0810	2022-08-10	29.2701	96.9439	4641	176.50	911	0.4284	0.05	36.54	N.A.	-16.20
	SETP_0911	2022-09-10	29.2701	96.9439	4641	176.50	911	0.0924	0.08	38.14	-650.9	-16.07
	SETP_1010	2022-10-10	29.2701	96.9439	4641	176.50	911	0.2069	0.00	50.71	-659.4	-16.41
	SETP_1101	2022-11-01	29.2701	96.9439	4641	176.50	911	0.2005	0.00	19.89	N.A.	-15.10

Table S2 Molecular composition of dissolved organic matter (DOM) in glacier outflow samples, including relative contributions of compound classes (based on formula classification) and intensity-weighted bulk chemical indices (O/C, H/C, N/C, S/C, AI_mod, NOSC, and molecular weight) derived from ultrahigh-resolution mass spectrometry. Percentages represent relative signal intensity contributions of assigned molecular formulas within each compound class.

sample name	Amino sugars (%)	Carbohydrates (%)	Condensed aromatics (%)	Lignin-like (%)	Lipid-like (%)	Peptide-like (%)	Aromatic-like (%)	Tannin-like (%)	Others (%)	O/C	H/C	N/C	S/C	AI_mod	NOSC	molecular weight	Formula number
ARJ	0.02	0.60	7.34	16.69	26.76	1.33	12.45	8.27	36.66	0.3154	1.3061	0.0187	0.0045	0.3157	-0.5325	250.1428	2489
DKMD	0.03	0.38	7.29	18.33	24.06	1.02	11.88	10.21	38.16	0.3315	1.2843	0.0161	0.0080	0.3139	-0.4821	261.4589	3151
HRQ	0.04	0.61	6.31	21.18	26.32	1.08	11.51	9.62	34.64	0.3131	1.2951	0.0161	0.0113	0.3137	-0.5199	255.4218	3278
LXZLB	0.05	0.46	7.37	24.73	11.10	1.11	13.10	13.89	42.14	0.3693	1.1910	0.0158	0.0019	0.3518	-0.3317	296.68919	5179
MK	0.02	0.42	6.14	18.51	31.52	0.93	11.69	7.30	32.37	0.2947	1.3208	0.0151	0.0030	0.3113	-0.6045	255.47375	2606
QY	0.01	1.57	4.56	13.09	37.38	2.37	7.49	5.62	35.50	0.3133	1.3995	0.0164	0.0035	0.2600	-0.6382	249.93023	2962
TLNS_4	0.01	0.44	5.06	21.94	23.07	1.79	8.83	7.70	40.68	0.3308	1.3065	0.0137	0.0020	0.3022	-0.5292	276.17356	4232
TLNS_6	0.01	0.48	5.61	20.07	28.17	1.41	9.36	8.84	36.76	0.3243	1.3240	0.0106	0.0040	0.2923	-0.5631	268.37373	3973
XJ_K	0.17	0.91	3.82	17.16	22.58	2.31	6.39	4.96	47.93	0.3718	1.3475	0.0237	0.0042	0.2851	-0.4672	287.50637	4236
XJ_Q	0.14	0.85	6.87	17.71	17.22	2.47	10.99	10.30	44.65	0.3640	1.2489	0.0288	0.0073	0.3266	-0.3480	277.54994	4638

sample name	Amino sugars (%)	Carbohydrates (%)	Condensed aromatics (%)	Lignin-like (%)	Lipid-like (%)	Peptide-like (%)	Aromatic-like (%)	Tannin-like (%)	Others (%)	O/C	H/C	N/C	S/C	AI_mod	NOSC	molecular weight	Formula number
YBJ	0.02	0.40	4.87	21.25	22.58	1.59	9.03	7.83	41.41	0.3431	1.3007	0.0170	0.0058	0.2981	-0.4803	272.33117	4112
YZF	0.01	0.34	4.66	21.15	26.76	1.34	7.87	7.04	39.07	0.3344	1.3151	0.0136	0.0073	0.2885	-0.5193	280.24883	3526
ZZG	0.06	0.19	8.58	19.54	36.68	1.12	10.77	8.48	24.82	0.2855	1.2972	0.0199	0.0147	0.3288	-0.5651	255.55388	2627
XC_0603	0.45	2.81	2.90	16.25	23.43	2.62	6.12	4.38	47.55	0.3492	1.3703	0.0211	0.0041	0.2651	-0.5221	266.0069	3331
XC_0620	0.17	1.98	2.90	15.88	24.38	2.74	6.12	4.13	47.79	0.3427	1.3668	0.0239	0.0042	0.2695	-0.5227	262.4025	3593
XC_0710	0.11	1.81	3.17	13.08	20.13	4.32	6.83	3.32	53.03	0.3560	1.3397	0.0347	0.0024	0.2767	-0.4434	271.6744	3794
XC_0730	0.12	1.96	4.02	11.46	26.44	4.09	7.01	3.40	47.61	0.3351	1.3703	0.0367	0.0026	0.2754	-0.5043	255.5582	3828
XC_0810	0.08	1.36	3.29	15.38	28.61	3.65	6.65	3.75	43.47	0.3215	1.3643	0.0276	0.0034	0.2814	-0.5552	259.2910	3740
ZF_0531	0.13	1.02	3.08	7.15	51.23	1.04	2.62	2.31	34.72	0.3113	1.5660	0.0119	0.0042	0.2167	-0.8313	225.5886	2173
ZF_0610	0.28	1.45	1.77	6.97	54.82	0.94	2.12	1.77	32.73	0.2935	1.5788	0.0091	0.0048	0.2004	-0.8782	222.3869	2015
ZF_0710	0.33	2.11	3.38	12.08	32.71	1.90	4.72	4.18	44.37	0.3388	1.4467	0.0182	0.0106	0.2382	-0.6196	251.4907	2843
ZF_0810	0.07	0.48	3.09	17.56	25.98	1.45	6.10	6.39	46.09	0.3575	1.3519	0.0149	0.0034	0.2785	-0.5449	277.8165	3792

sample name	Amino sugars (%)	Carbohydrates (%)	Condensed aromatics (%)	Lignin-like (%)	Lipid-like (%)	Peptide-like (%)	Aromatic-like (%)	Tannin-like (%)	Others (%)	O/C	H/C	N/C	S/C	AI_mod	NOSC	molecular weight	Formula number
ZF_0910	0.10	0.71	3.57	19.94	23.31	1.58	7.33	6.94	44.37	0.3544	1.3244	0.0179	0.0055	0.2891	-0.4872	281.4124	4022
ZF_1010	0.03	0.58	4.20	19.29	25.45	1.76	8.49	8.25	41.29	0.3373	1.3154	0.0168	0.0021	0.2990	-0.5181	272.1103	4106
SETP_0610	0.03	0.95	2.26	17.54	37.80	1.14	5.09	4.53	36.54	0.3152	1.4147	0.0091	0.0031	0.2512	-0.6804	267.3440	2785
SETP_0710	0.06	2.60	1.90	13.83	42.21	1.44	3.92	3.13	35.26	0.3190	1.4584	0.0082	0.0022	0.2230	-0.7174	266.8897	2500
SETP_0810	0.16	1.20	2.88	13.47	36.13	1.35	4.93	5.21	41.02	0.3270	1.4631	0.0103	0.0063	0.2343	-0.6908	251.6190	2992
SETP_0911	0.06	0.99	2.07	13.70	43.23	1.00	3.96	3.26	36.15	0.3069	1.4604	0.0074	0.0028	0.2251	-0.7445	260.5904	2528
SETP_1010	0.29	3.90	4.10	14.17	31.69	1.76	5.06	5.13	40.52	0.3550	1.4335	0.0155	0.0085	0.2477	-0.5945	264.1006	2742
SETP_1101	0.36	1.70	2.02	13.23	35.19	1.43	4.32	3.29	43.30	0.3286	1.4502	0.0123	0.0053	0.2258	-0.6699	256.5204	2722

Table S3-1. Exploratory single-predictor PERMANOVA analyses of associations between continuous environmental variables and DOM molecular composition in the spatial dataset.

variable	transformation	n	R^2	p value
Longitude	none	16	0.182567	0.0071
Latitude	none	16	0.041443	0.7849
Elevation	none	16	0.022132	0.994
Glacier area	log10	16	0.073211	0.3214
Vegetation cover	none	16	0.037765	0.8525
sRAD	none	16	0.183375	0.0079
Annual precipitation	none	16	0.078216	0.2743
Average Temperature (May- Oct)	none	16	0.065468	0.4128
δD	none	16	0.091308	0.1851
$\delta^{18}O$	none	16	0.08994	0.2045
DOC concentration	none	16	0.17712	0.0097
Conductivity	none	16	0.085443	0.2355
pH	none	16	0.036407	0.8649
Mg^{2+} (ng g^{-1})	log10	16	0.125558	0.0641
SO_4^{2-} (mg L^{-1})	none	16	0.113861	0.0954
Cl^- (mg L^{-1})	none	16	0.074215	0.3593
NO_3^- (mg L^{-1})	none	16	0.069736	0.3803
NH_4^+ (ng g^{-1})	log10	16	0.065593	0.4111
Na^+ (ng g^{-1})	log10	16	0.055223	0.5581
K^+ (ng g^{-1})	log10	16	0.04576	0.7062
Ca^{2+} (ng g^{-1})	log10	16	0.042804	0.7602

Notes: Each variable was evaluated separately using PERMANOVA based on Bray–Curtis dissimilarities calculated from Hellinger-transformed relative-intensity profiles of assigned molecular formulas in the spatial dataset (n = 16). Variables showing pronounced right-skewness or spanning a wide numerical range were $\log_{10}$-transformed as indicated in the table; all other variables were analyzed without transformation. Reported R^2 values represent marginal associations from separate single-predictor models and may include explanatory variation shared among correlated predictors; they should therefore not be summed or interpreted as independent effects. P-values were obtained using 9,999 permutations.

Glacier area was obtained from the Second Glacier Inventory Dataset of China (LIU et al., 2014). For sampling sites receiving outflow from multiple identifiable glacier termini, glacier areas were summed to represent the total contributing glacierized area. Vegetation cover was derived from ESA WorldCover land-cover fractions (Zanaga et al., 2022) within a 1-km-radius buffer around each spatial sampling site and was calculated as the sum of the moss and lichen, grassland, and tree-cover fractions. Surface downwelling shortwave radiation (sRAD), annual precipitation, and mean temperature from May to October were derived from the Third Pole

Meteorological Forcing Dataset (TPMFD; 1979–2023)(YANG et al., 2025). All other variables were measured in this study.

Table S3-2 Exploratory single-predictor PERMANOVA analyses of associations between categorical site characteristics and DOM molecular composition in the spatial dataset.

Predictor	Levels and sample sizes	PERMANOVA R^2	PERMANOVA p	Betadisper p
Glacier type	Continental, 5; Maritime, 1; Subcontinental, 10	0.17441	0.1826	NA
Rock type	Igneous, 4; Metamorphic, 11; Sedimentary, 1	0.169	0.2218	NA
Potential carbonate influence	Maybe, 5; No, 4; Yes, 7	0.09696	0.7891	0.324

Notes: Rock type and carbonatic classification were assigned to the best of our knowledge based on available geological maps and expert consultation. Because detailed site-specific lithological information and direct substrate verification were unavailable, these classifications should be regarded as approximate and may not fully represent the geological materials influencing each glacier outflow. In addition, the glacier-type and rock-type groupings contained singleton categories and highly unequal group sizes; their PERMANOVA results should therefore be considered exploratory, and multivariate dispersion was not evaluated for these two predictors.

Table S4 Variation partitioning of DOM molecular composition among longitude, shortwave radiation, and annual precipitation across the spatial dataset.

Model or fraction	Adjusted R^2	p
Full model: longitude + radiation + precipitation	0.1366	0.032
Unique longitude	0.0327	0.157
Unique shortwave radiation	0.0221	0.221
Unique annual precipitation	−0.0074	0.480
Shared longitude–radiation	0.0694	—
Shared longitude–precipitation	−0.0138	—
Shared radiation–precipitation	−0.0023	—
Shared among all three predictors	0.0359	—

Note: Variation partitioning was based on Bray–Curtis dissimilarities calculated from Hellinger-transformed molecular-intensity data. Adjusted R^2 values are reported. Significance was tested for the full model and unique fractions using permutation tests. Shared fractions cannot be tested independently. Negative adjusted fractions were retained in the calculations but were not interpreted ecologically.

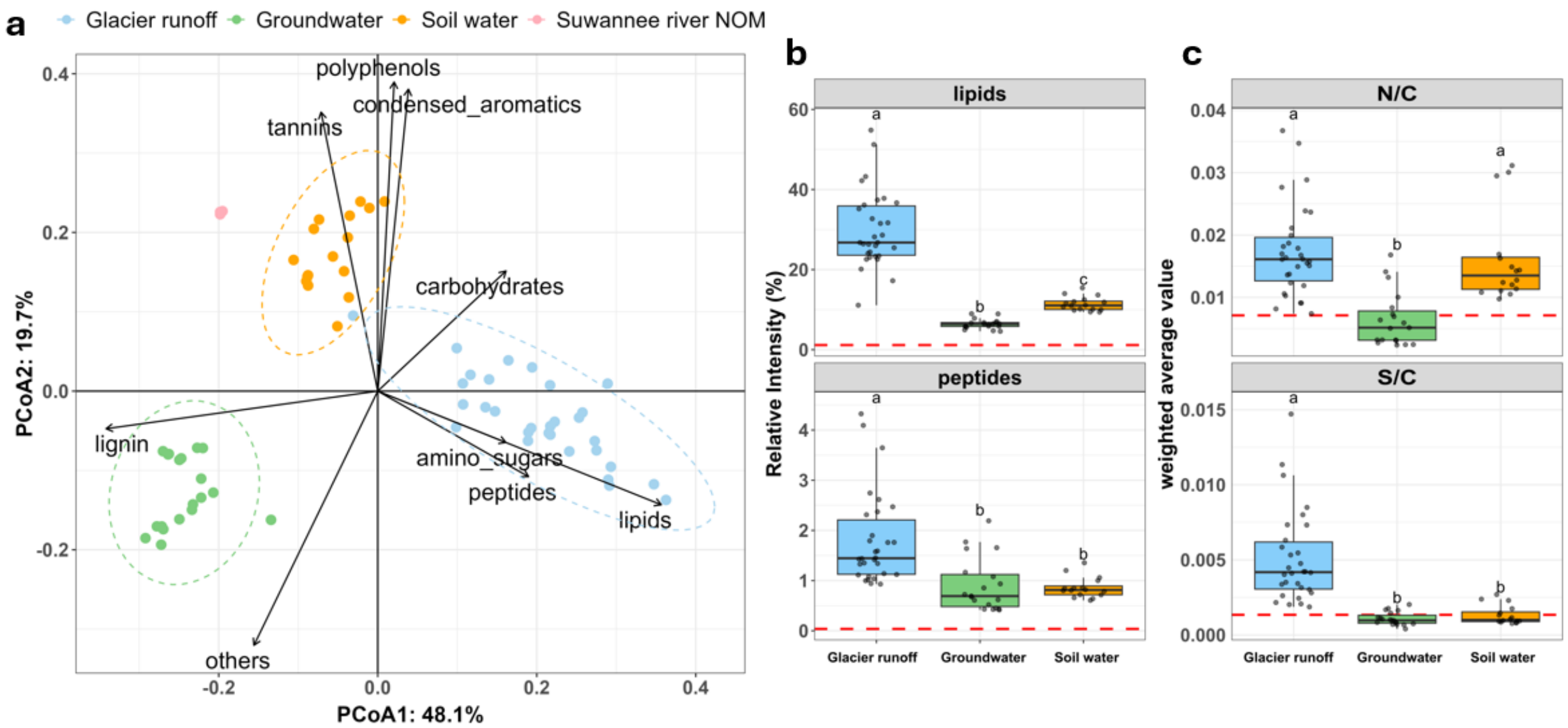

Figure S1. Comparison of glacier runoff DOM molecular composition from this study with other terrestrial aquatic DOM to assess the distinctiveness of glacier runoff DOM and its potential relevance for recipient ecosystems. Reference datasets included groundwater DOM from two contrasting geological settings in central Germany, namely carbonate bedrock and sandstone bedrock, which exhibit distinct molecular compositions (Benk et al., 2019; Schroeter et al., 2025); Suwannee River natural organic matter (SRNOM); and soil-water DOM collected during snowmelt events from lysimeters installed in forest and grassland sites (Huang et al., 2024). (a) Principal coordinates analysis (PCoA) based on Bray–Curtis dissimilarity of DOM molecular composition across the different terrestrial aquatic systems. Vectors indicate fitted molecular-group variables based on summed relative intensities. (b) Summed relative intensities of major molecular groups across sample types. (c) Intensity-weighted bulk DOM indices across sample types. Red dotted lines in panels (b) and (c) indicate the mean values of the corresponding molecular groups or bulk indices in SRNOM.

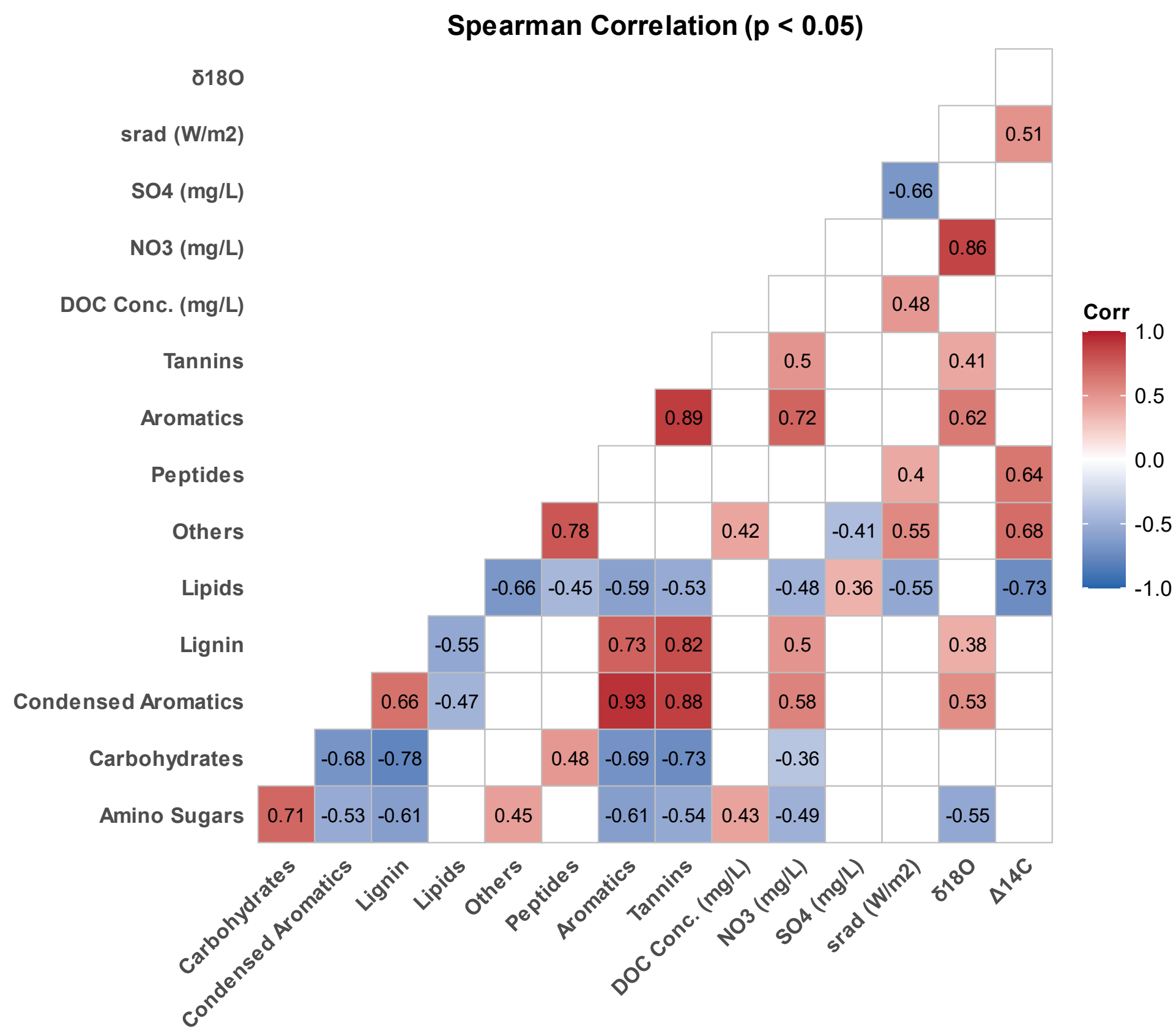

Figure S2. Spearman correlation matrix showing pairwise relationships (ρ) between inorganic solutes, isotopic indicators, and environmental variables and molecular-level DOM compound-class contributions (summed relative intensity) in glacier outflow samples. Colors indicate ρ (positive to negative); symbols denote statistically significant correlations ($p < 0.05$).

Correlations were calculated using pairwise complete observations; sample size n = 30 for all variables except for $\Delta^{14}C$, where n=23.

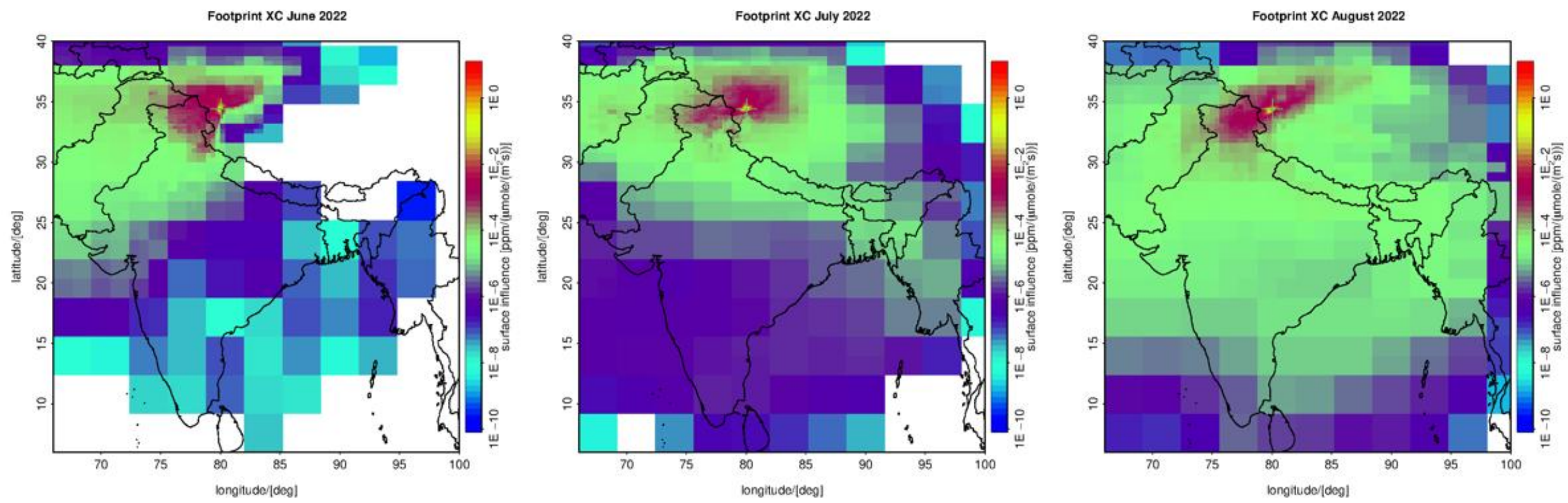

Figure S3. Monthly mean STILT footprint fields for the XC sampling site (June –August 2022). Footprints indicate the potential influence of upwind regions on the sampling site, with warmer colors representing stronger potential contributions. June values represent simulations from 10–30 June only.

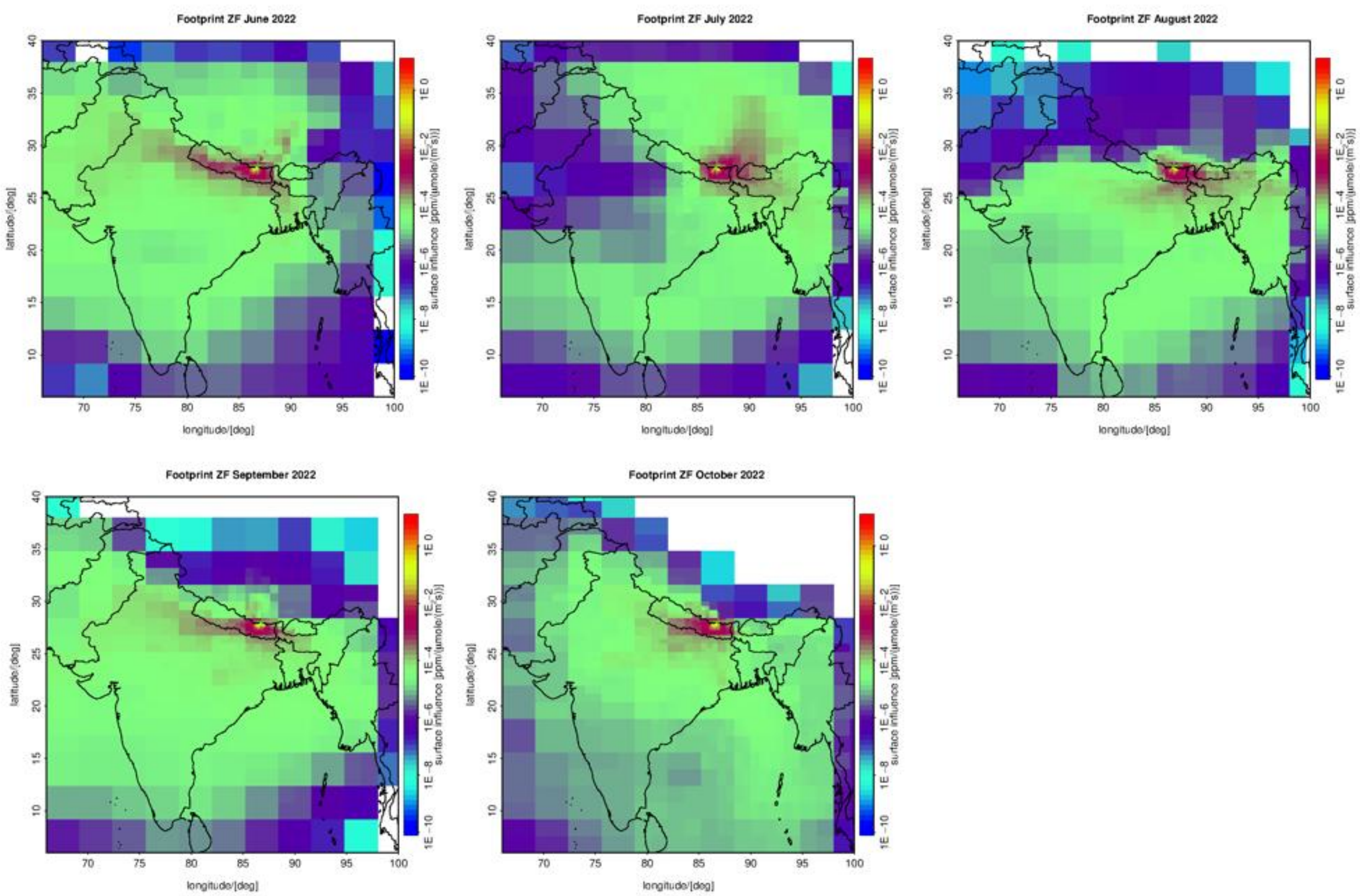

Figure S4. Monthly mean STILT footprint fields for the ZF sampling site (June– October 2022). Footprints indicate the potential influence of upwind regions on the sampling site, with warmer colors representing stronger potential contributions. June values represent simulations from 10–30 June only.

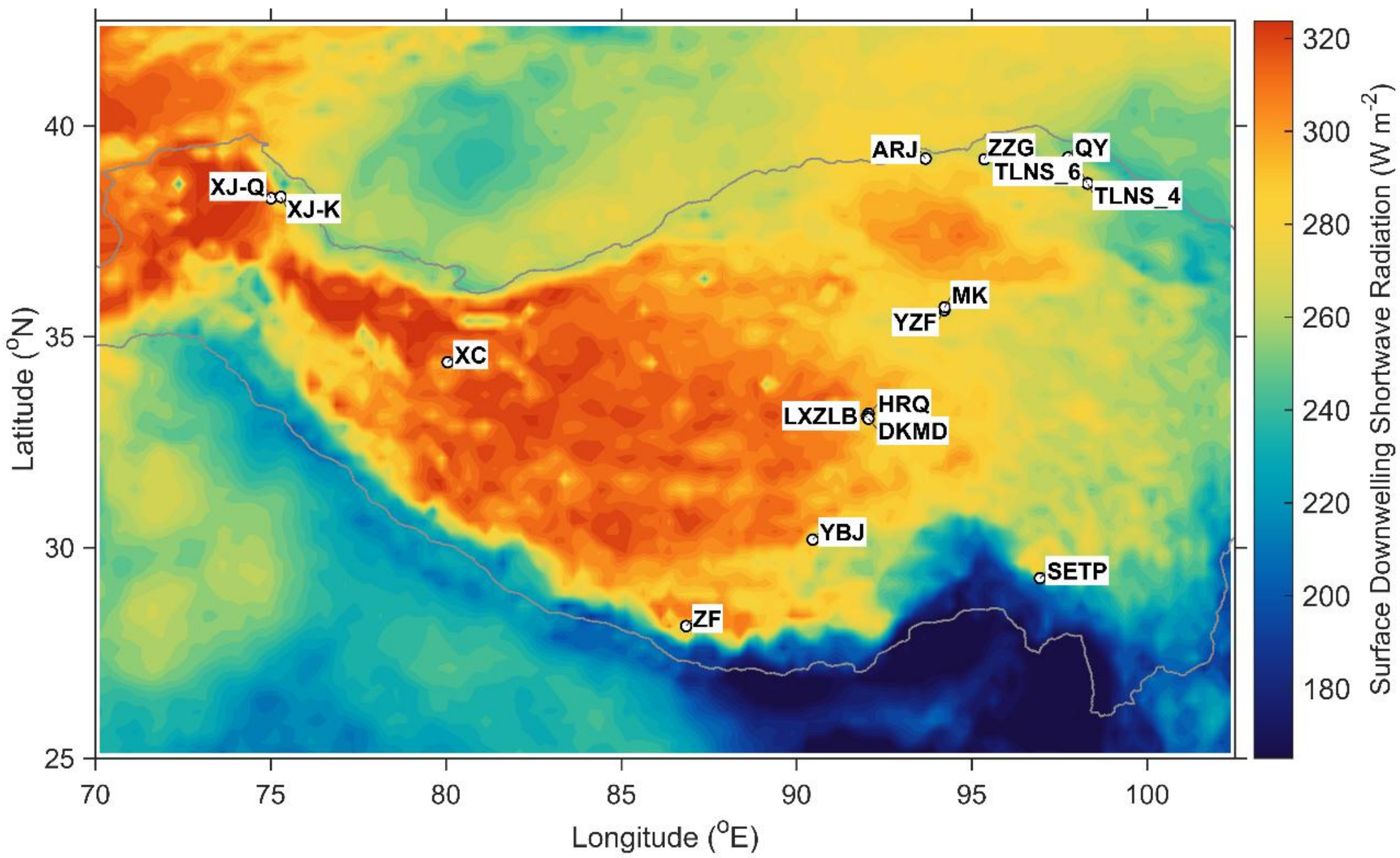

Figure S5 Spatial distribution of surface downwelling shortwave radiation (W m-2) during June–August 2022. The radiation field represents the monthly mean surface downwelling shortwave radiation at a spatial resolution of 0.25° and was derived from the CM SAF CLARA-A3 satellite product available through the Copernicus Climate Data Store (Copernicus Climate Change Service, 2020)

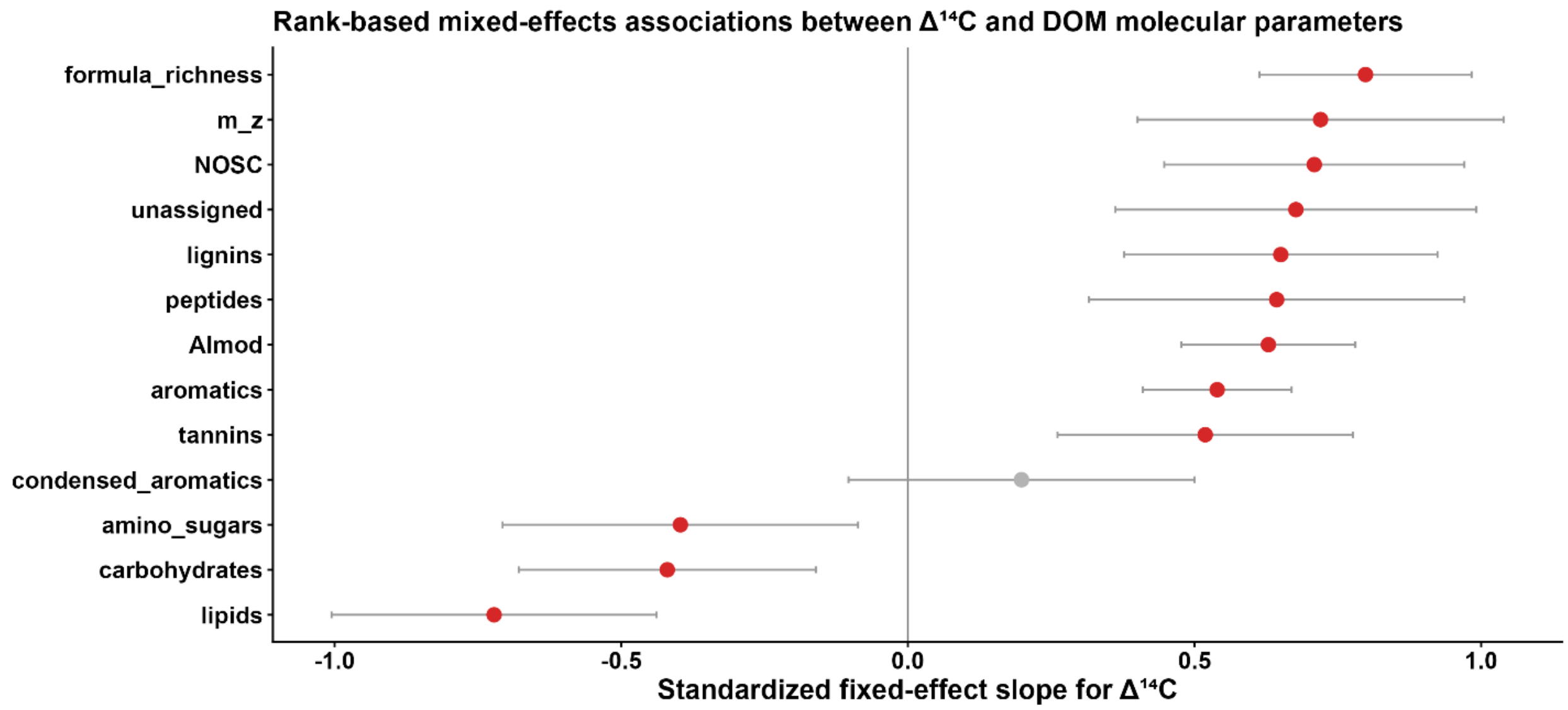

Figure S6. Associations between $\Delta^{14}C$ and DOM molecular parameters evaluated using rank-based linear mixed-effects models with glacier identity included as a random intercept. Points represent standardized fixed-effect slopes, and horizontal lines show 95% confidence intervals. Red points indicate relationships significant after Benjamini–Hochberg correction ($P_{adj}<0.05$); gray points indicate nonsignificant relationships.

Reference:

Benk, S. A., Yan, L., Lehmann, R., Roth, V.-N., Schwab, V. F., Totsche, K. U., et al. (2019). Fueling Diversity in the Subsurface: Composition and Age of Dissolved Organic Matter in the Critical Zone. *Frontiers in Earth Science*, *7*, 296. https://doi.org/10.3389/feart.2019.00296

Copernicus Climate Change Service. (2020). Surface radiation budget from 1982 to present derived from satellite observations [Data set]. ECMWF. https://doi.org/10.24381/CDS.CEA58B5A

Huang, C., Schroeter, S. A., Lehmann, K., Herrmann, M., Totsche, K. U., & Gleixner, G. (2024). Snowmelt seepage fluxes of dissolved organic matter in forest and grassland – a molecular-level case study from the Hainich Critical Zone Exploratory, Germany. *Frontiers in Earth Science*, *12*, 1458322. https://doi.org/10.3389/feart.2024.1458322

LIU, S., GUO, W., & XU, J. (2014). The second glacier inventory dataset of China (version 1.0) (2006-2011) [Data set]. National Tibetan Plateau Data Center. https://doi.org/10.3972/glacier.001.2013.db

Schroeter, S. A., Orme, A. M., Lehmann, K., Lehmann, R., Chaudhari, N. M., Küsel, K., et al. (2025). Hydroclimatic extremes threaten groundwater quality and stability. *Nature Communications*, *16*(1), 720. https://doi.org/10.1038/s41467-025-55890-2

YANG, K., JIANG, Y., TANG, W., HE, J., SHAO, C., ZHOU, X., et al. (2025). A high-resolution near-surface meteorological forcing dataset for the Third Pole region （TPMFD, 1979-2023） [Data set]. National Tibetan Plateau Data Center. https://doi.org/10.11888/Atmos.tpdc.300398

Zanaga, D., Van De Kerchove, R., Daems, D., De Keersmaecker, W., Brockmann, C., Kirches, G., et al. (2022). ESA WorldCover 10 m 2021 v200. https://doi.org/10.5281/zenodo.7254221
